# Different results despite high homology: Comparative expression of human and murine DNase1 in *Pichia pastoris*

**DOI:** 10.1371/journal.pone.0321094

**Published:** 2025-04-29

**Authors:** Jan-Ole Krischek, Hans Georg Mannherz, Markus Napirei

**Affiliations:** 1 Department of Anatomy and Molecular Embryology, Medical Faculty, Ruhr-University Bochum, Bochum, Germany; 2 Department of Cellular and Translational Physiology, Medical Faculty, Ruhr-University Bochum, Bochum, Germany; Leibniz-Institut fur Naturstoff-Forschung und Infektionsbiologie eV Hans-Knoll-Institut, GERMANY

## Abstract

The prolonged persistence of extracellular chromatin and DNA is a salient feature of diseases like cystic fibrosis, systemic lupus erythematosus and COVID-19 associated microangiopathy. Since deoxyribonuclease I (DNase1) is a major endonuclease involved in DNA-related waste disposal, recombinant DNase1 is an important therapeutic biologic. Recently we described the production of recombinant murine DNase1 (rmDNase1) in *Pichia pastoris* by employing the α-mating factor prepro signal peptide (αMF-SP) a method, which we now applied to express recombinant human DNASE1 (rhDNASE1). In addition to an impaired cleavage of the αMF pro-peptide, which we also detected previously for mDNase1, expression of hDNASE1 resulted in a 70–80 times lower yield although both orthologues share a high structural and functional homology. Using mDNase1 expression as a guideline, we were able to increase the yield of hDNASE1 fourfold by optimizing parameters like nutrients, cultivation temperature, methanol supply, and codon usage. In addition, post-translational import into the rough endoplasmic reticulum (rER) was changed to co-translational import by employing the signal peptide (SP) of the α-subunit of the Oligosaccharyltransferase complex (Ost1) from *Saccharomyces cerevisiae*. These improvements resulted in the purification of ~ 8 mg pure mature rmDNase1 and ~ 0.4 mg rhDNASE1 per Liter expression medium of a culture with a cell density of OD_600_ =  40 in 24 hours. As a main cause for the expression difference, we assume varying folding abilities to reach a native conformation, which induce an elevated unproductive unfolded protein response within the rER during hDNASE1 expression. Concerning functionality, rhDNASE1 expressed in *P. pastoris* is comparable to Pulmozyme®, i.e. rhDNASE1 produced in Chinese hamster ovary (CHO) cells by Roche - Genentech. With respect to the biochemical effectivity, rmDNase1 is superior to rhDNASE1 due to its higher specific activity in the presence of Ca^2 + ^/Mg^2 +^ and the lower inhibition by monomeric actin.

## Introduction

Purified or recombinant proteins like DNase1 - a waste disposal nuclease for chromatin and DNA of dying cells and neutrophil extracellular traps (NETs) [1–4] - are of interest as human therapeutics [5]. Discovered as a digestive enzyme of the pancreas by McCarty in 1946 [6] and isolated by Kunitz in 1950 [7], purified bovine (b)DNASE1 (Dornavac) obtained approval for human use in the USA in 1958 to liquefy purulent mucus in chronic bronchitis and cystic fibrosis (CF) [8–10]. In 1993, Genentech developed recombinant human (rh)DNASE1 (Dornase alfa, Pulmozyme®) as a physiological alternative, because purified bDNASE1 appeared immunogenic and contained impurities [11,12]. Thus Pulmozyme® beside Humulin® (human insulin) is one of the oldest biologics known [5].

Further situations necessitating chromatin and DNA disposal exist like the prevention of antinuclear autoimmunity causing systemic lupus erythematosus with lupus nephritis. Both, DNase1 and DNase1-like 3 (DNase1L3), eliminate these autoantigens that might otherwise induce pathogenic anti-dsDNA antibodies [3,4,13–19] and they clean glomerular capillaries from DNA containing immune deposits [16,20,21]. Furthermore, degradation of chromatin released as NETs prevents vascular microthrombi [2], which becomes particularly necessary during sepsis and COVID-19 [22–24]. A reduced NET degradation is not only characteristic for the pulmonary mucus in CF [25], but also for the tear film during dry eye disease [26,27], and as a side effect of tumors [28]. Additionally, removal of chromatin released from cardiomyocytes during infarction improves ventricular remodeling [29].

**Fig 1:**
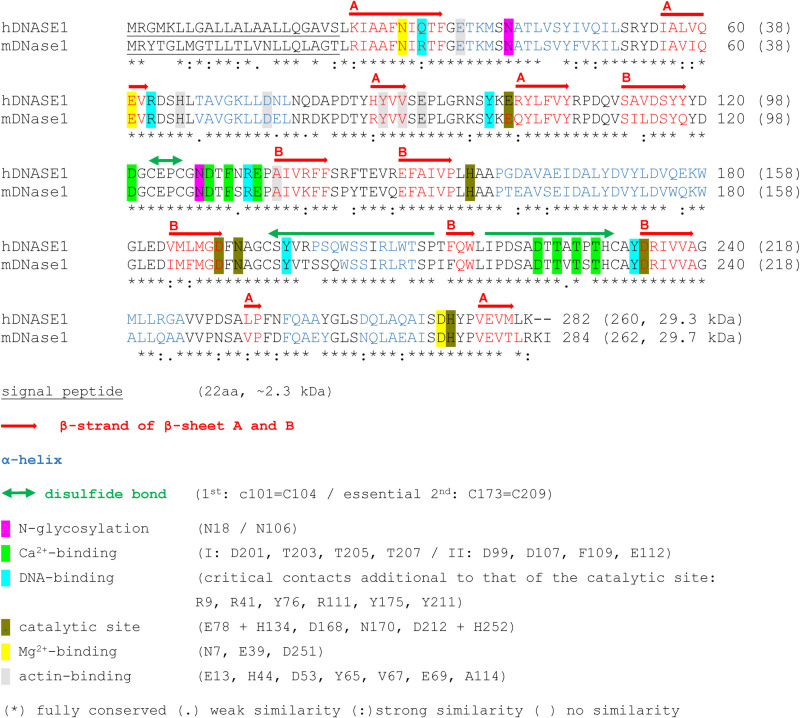
Comparison of human and murine DNase1. Protein sequence alignment performed by Clustal Omega (EMBL-EBI). The amino acid sequence lengths of pre- and mature DNase1 are indicated. Secondary protein structures for hDNASE1 are taken from the 3D structure databases of UniProt (Acc. No. P24855), in detail from the Protein Data Bank in Europe based on X-ray analysis (PDBe: 4awn) [36]. The data for mDNase1 are from UniProt (Acc. No. P49183) and predicted by the SWISS-MODEL Repository based on hDNASE1. The DNase1 protein possesses a four-layered αβ-sandwich structure with two central β-sheets each composed of six β-strands surrounded by overall ten α-helices [35–37]. The comparison of functional amino acids concerning disulfide bridges, Ca^2 + ^-binding [35,39,40], N-glycosylation [41–43], DNA-binding [44–46], catalytic center, Mg^2 + ^-binding [36,44,45,47], and actin-binding [48,49] indicates a high conservation between human and mDNase1.

Functionally DNase1 is a sequence unspecific endonuclease hydrolyzing dsDNA in the presence of appropriate divalent cations at pH 7–8 [30]. In contrast to DNase1L3, which cleaves protein-free DNA with low and chromatin at internucleosomal sites with high efficiency, DNase1 acts opposite [31,32] and cooperates with proteases like plasmin to degrade chromatin [1]. Since DNase1 occurs in most mammalian body fluids [33], it has a proteolytic resistance given by two N-glycosylations and two Ca^2 + ^-ions, which protect the molecular surface [34,35], and a high conformational stability, which arises from a four-layered αβ-sandwich structure with two disulfide bridges and two central β-sheets each composed of six β-strands surrounded by overall ten α-helices [35–37]. When comparing DNase1 orthologues it becomes obvious that their structure and main functional regions are highly conserved [38] (Fig 1).

Besides its isolation from salivary glands [7,37,50,51], DNase1 was initially produced in *E. coli* under the control of an inducible promoter because it acted cytotoxic [52]. Although N-glycosylation shall influence its specific activity [34], non-glycosylated bDNASE1 produced by *E. coli* was evaluated equal to that purified from pancreas [52,53]. To ensure the natural glycosylation and proper folding, DNase1 was mostly transiently or stably inducible expressed in eukaryotic cells like human embryonic kidney 293, HeLa S3 [54], or CHO cells [55,56] as well as NIH-3T3 fibroblasts [31] with or without a purification tag. Beside mammalian also yeast cells came into the focus of interest. Thus, Worthington produces rbDNASE1 for molecular biological applications in *P. pastoris*. Furthermore, the members of the murine (m)DNase1 family and hDNASE1L2 were expressed in *P. pastoris* [57,58]. Remarkably, no reports exist about expression of rhDNASE1 in yeast. Major advantages of yeast are low culture costs, a short reproduction time, and a prospect of a high protein yield [59]. Disadvantageous is the inherent N-glycosylation of the high-mannose type [60], which can lead to a fast degradation or an immune reaction when biologics are administered to mammals [60,61]. However, progress was made to humanize the glycosylation like, e.g., for erythropoietin or IgG antibodies [62–64]. Unfortunately, protein expression in yeast can be accompanied by an inefficient protein folding leading to an unfolded protein response (UPR) by accumulated proteins within the rER. As a result, the transmembrane kinase with cytoplasmic endonuclease activity called Ire1p is activated, which together with the tRNA ligase Rlg1p splices the *Hac1* mRNA to functional *Hac1s*. [65,66]. Under favorable conditions, the transcription factor Hac1 induces chaperons to improve protein folding, but can also trigger rER-associated protein degradation [65–68] or -autophagy [69,70] when improvement fails.

Recombinant secretory protein production in yeast often employs the αMF-SP. The αMF is a 13 amino acid long pheromone regulating conjugation, which is generated from a multimeric precursor encoded by the *MF(ALPHA)1* gene [71,72]. The αMF-SP consists of a 19 amino acid long pre-peptide facilitating translocation of αMF into the rER and a 66 amino acid long, tri-N-glycosylated pro-peptide mediating its transfer to the Golgi apparatus (GA) via an Erv29p dependent packaging into COPII vesicles [73,74]. The pre-peptide is removed by a signal peptidase of the rER, whereas the pro-peptide is cleaved by Kex2 within the GA. An EAEA tetrapeptide follows to the pro-peptide and is trimmed by Ste13 within the GA too [72,74]. Notably, the secretory pathway of yeast can be dependent or independent on a signal recognition particle (SRP) [64]. The SRP-dependent pathway acts co-translationally with the SRP-receptor and the translocon Sec61p within the rER membrane, whereas the independent pathway acts post-translationally via Sec62/63p plus Sec61p [75]. The αMF-SP works SRP-independent [76] and its post-translational rER import is supported by the pro-peptide [77]. Despite the wide use of the αMF-SP, problems can occur like an inefficient cleavage of the pro- or tetrapeptide leading to N-terminal protein elongations [60,77]. The αMF-SP in the expression vector pPinkα-HC used in our previous and actual study is devoid of the tetrapeptide (S1 Fig), however hyper-tri-N-glycosylated pro-peptide extensions indeed occurred during expression of the mDNase1 family in *P. pastoris* [57]. Another problem with the αMF-SP can be an impaired post-translational rER import of proteins exhibiting a strong self-folding ability in the cytoplasm [77].

The *PichiaPink™* expression system works with methylotrophic *P. pastoris* of the Mut^ +^ genotype, which can oxidize methanol (MeOH) to formaldehyde by the alcohol oxidases AOX1 and AOX2 [78,79]. Formaldehyde subsequently enters anabolic or catabolic pathways [60], which guarantees cell survival in the absence of further carbon sources. In addition, MeOH induces transgene expression via the AOX1 promoter as part of the vectors used [78]. Employing the aforementioned system, we here describe a comparative expression and biochemical characterization of hDNASE1 and mDNase1.

## Materials and methods

### Materials and equipment

Amicon: 3K centrifugal filters. Beckman: DJ640 spectrophotometer. Berten Technologies: homogenizer Precellys® 24. BioRad: Gel-imager ChemiDoc™ XRS + System with the ImageLab 5.0 software, Bio-Gel HT hydroxyapatite, Bio-Gel P-100, Protein Assay Dye Reagent. Calbiochem: calf thymus DNA. Eurofins: primers. Eppendorf: Electroporator 2510. Foremedium: Kaiser’s SC-Ade mixture, peptone, yeast nitrogen base (YNB), yeast extract. Invitrogen: *PichiaPink™* expression system with vector pPinkα-HC. KAPA Biosystems: KAPA HiFi HotStart ReadyMix. Macherey Nagel: NucleoSpin RNA Mini Kit. Millipore: 0.45 µm filter units. New Brunswick: Incubator Innova 42. New England Biolabs: ProtoScript® II reverse transcriptase, Remove-iT® PNGase F, restriction enzymes. PAN-Biotech: Prestained protein marker. Roche: EDTA-free protease inhibitor cocktail cOmplete™ ULTRA, Pulmozyme®. SERVA: Coomassie brilliant blue G-250. Sigma-Aldrich: biotin, chloramphenicol, protease inhibitor cocktail. ThermoScientific: AEBSF, PageRuler™ Prestained Protein Ladder, 5K centrifugal filters. Whatman BioSystems: DEAE cellulose DE53.

### Sequence references

Nucleotide sequence for *Mus musculus* DNase1 (NCBI Acc. No. **NM_010061**), *Homo sapiens* DNASE1 (NCBI Acc. No. **NM_005223.4**), and for the α-subunit of the **O**ligo**s**accharyl**t**ransferase complex (Ost1) of *S. cerevisiae* (NCBI Acc. No. **NM_001181436.3**). Protein sequence for *M. musculus* DNase1 (UniProtKB Acc. No. **P49183**), *H. sapiens* DNASE1 (UniProtKB Acc. No. **P24855)**, and for Ost1 of *S. cerevisiae* (UniProtKB Acc. No. **P41543**). Vector pPinkα-HC (SnapGene vector databank, sequence author: Thermo Fisher - Invitrogen).

### Vector constructions for murine DNase1 expression

The vector pPinkα-HC/αMF-mDNase1 for expression of mDNase1 with the entire αMF-SP was cloned as described [57]. Using the recently modified vector pPinkHC (S1 File) [57], we cloned the pPinkHC/preαMF-mDNase1 (S2 File) and -mDNase1^A114Y^ vectors lacking the αMF pro-peptide. Therefore, the *pre*α*MF-mDNase1* or *-mDNase1*^*A114Y*^ cDNA were amplified using primers preαMF-mD1-for and mD1-rev and cloned between the *Stu*I - *Kpn*I sites of pPinkHC. As a PCR template pDs/mDNase1 or pDs/mDNase1^A114Y^ were employed [80]. A second expression cassette was added by amplifying the existing cassette using Primers AOX1-for2 and CYC1-rev2. Subsequently, the second cassette was cloned into the *Aat*II site of the corresponding vectors generating pPinkHC/preαMF-mDNase1 2x and -mDNase1^A114Y^ 2x (S2 File). Both cassettes had the same transcription direction. To evaluate DNase1 cytotoxicity in *P. pastoris* an inactivating D168S point mutation in the catalytic center [47] was introduced into the pPinkHC/preαMF-mDNase1 vector by generating two amplicons using primers preαMF-mD1-for and mD1^D168S^-rev or mD1^D168S^-for and mD1-rev. Both amplicons were cut with *Pst*I, ligated, cut with *Kpn*I, and finally cloned between the *Stu*I - *Kpn*I sites of pPinkHC to generate pPinkHC/preαMF-mDNase1^D168S^. To evaluate the influence of post- vs. co-translational import, vector pPinkHC/preOst1-mDNase1 employing the Ost1-SP for co-translational import into the rER was generated [81,82]. Therefore, the *preOst1-mD1* cDNA was amplified from pDs/mDNase1 using primers preOst1-mD1-for and mD1-rev and the amplicon was cloned into the *Stu*I - *Kpn*I sites of pPinkHC. A second expression cassette was included as described above creating pPinkHC/preOst1-mDNase1 2x. Primers are listed in Table 1.

**Table 1:** Primers.

cloning primers	
name	sequence and cloning site
preαMF-mD1-for	5‘GAAACGATGAGATTTCCTTCAATTTTTACTGCTGTTTTATTCGCAGCATCCTCCGCATTAGCTCTGAGAATTGCAGCCTTCAACATTCGG-3‘ blunt-end cloning into StuI site
mD1-rev	5’-AACTGCTCAGGTACCTCAGATTTTTCTGAGTG-3’ KpnI cloning
mD1^D168S^-for	5´-GGATCTTTCAATGCTGGCTGCAGCTACGTC-3´ PstI cloning
mD1^D168S^-rev	5´-GACGTAGCTGCAGCCAGCATTGAAAGATCC-3´ PstI cloning
preOst1-mD1-for	5´GAAACGATGAGGCAGGTTTGGTTCTCTTGGATTGTGGGATTGTTCCTATGTTTTTTCAACGTGTCTTCTGCTCTGAGAATTGCAGCCTTCAACATTCGG-3‘ blunt-end cloning into StuI site
hD1-for	5’-CTGAAGATCGCAGCCTTCAACATCC-3’ blunt-end cloning into StuI site
hD1^K2R^-for	5’-CTGAGGATCGCAGCCTTCAACATCC-3’ blunt-end cloning into StuI site
hD1-rev	5’-GTATCGGTACCCTTTCACTTCAGCATCACC-3’ KpnI cloning
preαMF-hD1-for	5´AAACGATGAGATTTCCTTCAATTTTTACTGCTGTTTTATTCGCAGCATCCTCCGCATTAGCTCTGAAGATCGCAGCCTTCAACATCC-3‘ blunt-end cloning into StuI site
hD1^D168S^-for	5´-GGCTCTTTCAATGCGGGCTGCAGCTATGTGA-3´ PstI cloning
hD1^D168S^-rev	5´-TCACATAGCTGCAGCCCGCATTGAAAGAGCC-3´ PstI cloning
preOst1-hD1-for	5´GAAACGATGAGGCAGGTTTGGTTCTCTTGGATTGTGGGATTGTTCCTATGTTTTTCAACGTGTCTTCTGCTCTGAAGATCGCAGCCTTCAACATCC-3‘ blunt-end cloning into StuI site
AOX1-for2	5’-CCCCCTCGACGTCTAACATCCAAAGACGAAAGG-3’ AatII cloning
CYC1-rev2	5’-CCGCGGTGACGTCGGCAAATTAAAGCCTTCGAGCG-3’ AatII cloning
primers for sequencing and RT-PCR	
name	sequence
AOX1-for1	5‘-GACTGGTTCCAATTGACAAGC-3‘
CYC1-rev1	5‘-GTCACGCTTACATTCACGC-3‘
preαMF-mhD1-for	5‘-GTTTTATTCCAGCATCCTCC-3‘
mhD1-for	5‘-GTACAGGCCTGACCAGGTGTC-3‘
mhD1-rev	5‘-GGGATCACCACTGGAAG-3‘
Hac1-for1	5‘-GAAACGAGGCCTSAAACGATCCCGTAGATTCTTCTC-3‘
Hac1-rev1	5‘-CCGGCCGGTACCCTATTCCTGGAAGAATACAAAGTC-3‘
Hac1-rev2	5‘-TAAATGGCCGGCCGGTACCCTATTCC-3‘
Act1-for	5‘-GGTGTCATGGTCGGTATGGG-3‘
Act1-rev	5‘-CTACCCAAGTCGATACG-3‘

### Vector constructions for human DNASE1 expression

The *hDNASE1* cDNA and its codon optimized (co) form (Jan-Ole Krischek, S3 File) were supplied from GenScript in pBSIIKS(+)-hDNASE1 and -hDNASE1^co^. For expression of mature hDNASE1 with the αMF-SP, its cDNA was amplified using primers hD1-for/-rev and cloned into the *Stu*I - *Kpn*I sites of pPinkα-HC to generate vector pPinkα-HC/αMF-hDNASE1. The same was done for mature hDNASE1 possessing an N-terminus adapted to mDNase1 by introducing a K2R mutation. To generate pPinkHC/preαMF-hDNASE1^K2R^, primers hD1^K2R^-for and hD1-rev were used. Cloning of pPinkHC/preαMF-hDNASE1 and -hDNASE1^co^ lacking the αMF pro-peptide as well as pPinkHC/preαMF-hDNASE1^co^ 2x with two expression cassettes occurred analogous to the murine vectors. The same applied for pPinkHC/preαMF-hDNASE1^D168S^ coding for inactive hDNASE1 and for pPinkHC/preOst1-hDNASE1^co^ 1x and 2x. Primers are listed in Table 1.

### Transformation of *PichiaPink*^*TM*^ *pastoris*

Transgenic *PichiaPink™ pastoris* cells were generated as described [57]. In brief, cells were streaked and incubated on YPD plates (1% (w/v) yeast extract, 2% (w/v), peptone, 2% (w/v) dextrose, 2.4% (w/v) agar) at 28 °C and a starter culture was established with a single colony (Incubator Innova 42). For electroporation, 100 mL YPD culture of OD_600_ =  1.3–1.5 was washed twice with ice-cold water and resuspended twice in 1 M sorbitol. About 5 µg *Afl*II linearized vector was mixed with 80 µL cells in a cuvette and transformation was done at 1,500 V using Electroporator 2510. Afterwards cells were incubated in 1 mL YPD with 1 M sorbitol for 2 hours at 26 °C without shaking. Aliquots were spread onto Pichia adenine drop-out plates (100 mM potassium phosphate buffer (PPB), pH 6, 0.2% (w/v) Kaiser’s SC-Ade mixture, 1.34% (w/v) YNB, 2% (w/v) dextrose, 2.4% (w/v) agar) and incubated at 28 °C. Single white colonies were restreaked on plates and liquid starter cultures using synthetic complete dextrose adenine drop-out medium (SCD-Ade: 100 mM PPB, pH 6, 0.2% (w/v) SC-Ade mixture, 2% (v/v) dextrose, 1.34% (w/v) YNB, 0.0004% (w/v) biotin, 25 µg/mL chloramphenicol) were prepared. Transformation was verified by transgene specific PCR.

### Growth and expression cultures

#### Low scale culture.

Most experiments were done as 5–10 mL expression cultures for each setting. For biomass production, 100–500 mL growth medium was inoculated with a starter culture to an OD_600_ =  0.05 in a 1–5 L baffled flask (ratio of culture to flask volume 1:10) and incubated with shaking at 250 rpm and 24 or 28 °C until reaching an OD_600_ =  6. Growth culture media varied: we tested 0.25–1x buffered glycerol complex medium (BMGY) with respect to the amount of peptone and yeast extract (1x BMGY: 100 mM PPB, pH 6, 1% (w/v) yeast extract, 2% (w/v) peptone, 1% (v/v) glycerol, 1.34% (w/v) YNB, 0.0004% (w/v) biotin, 25 µg/mL chloramphenicol) or synthetic complete glycerol adenine drop-out (SCG-Ade) medium (SCD-Ade with 1% (v/v) glycerol instead of dextrose). After growth, cells were sedimented at 1,500 g, washed with PBS, pH 6 for 30 minutes at room temperature, sedimented again, suspended in 5–10 mL expression medium with an OD_600_ =  60 in 50–100 mL non-baffled flasks (reduced cell adhesion at the flask) and incubated with shaking at 175 rpm and 24 or 28 °C. Expression media varied: we tested 0.5–2% (w/v) peptone medium (PM) with respect to the peptone amount (100 mM MES-buffer, pH 6, 0.5–2% (w/v) peptone, 1.34% (w/v) YNB, 0.0004% (w/v) biotin, 25 µg/mL chloramphenicol) or SCM (SC-Ade medium with MeOH instead of dextrose or glycerol). Experiments were performed with or without MeOH. After expression, cells and debris were sedimented at 1,500 and 14,000 g, respectively. Expression culture supernatant (SN) was employed in assays.

#### Large scale culture.

For purification of DNase1, 0.5–1 L 0.5x BMGY growth medium was employed. Cells were grown to OD_600_ =  6 with shaking at 250 rpm and 28 °C in 5 L baffled flasks, washed with PBS, pH 6 as described above, and transferred at an OD_600_ =  40 into 1% (w/v) PM containing 2.5% (v/v) MeOH. Expression was performed for 24 hours with shaking at 175 rpm and 28 °C. The culture SN was collected by cell sedimentation at 1,500 g and cell debris removed by filtering through a 0.45 µm filter unit.

### RNA extraction and RT-PCR

Total RNA of 80 mg cells was isolated with the NucleoSpin RNA Mini Kit. About 0.1 µg RNA was transcribed using ProtoScript® II reverse transcriptase (RT) according to the manufacturer`s protocol. For each gene-specific PCR, 1 µL RT-reaction was employed in 25 µL KAPA HiFi HotStart ReadyMix to detect *DNase1*, *Act1,* and *Hac1s* mRNA (Primers: Table 1). All PCRs were performed with the same protocol running 30 cycles with 15 seconds at 98 °C, 30 seconds at 58 °C, and 30 seconds at 72 °C. Of each PCR-sample 5 µL were investigated by TAE-agarose gel-electrophoresis.

### Cell extracts

About 100 mg of cells resuspended in 1 mL of radioimmunoprecipitation lysis buffer (RIPA) containing protease inhibitor cocktail were mechanically lysed with glass beads using the homogenizer Precellys® 24 at 5,000 rpm for three times and each 20 seconds. Thereafter, two cycles of freezing to − 80 °C and thawing were applied. Cell debris and glass beads were sedimented at 11,000 g for 10 minutes.

### Protein quantification

Protein concentrations were determined by a Bradford assay [83] using Protein Assay Dye Reagent according to the protocol published on the Bio-Rad website. Bovine serum albumin served as a standard. Protein concentration of purified recombinant DNase1 was proven or counter calculated by de-N-glycosylation performing SDS-PAGE, silver staining, and densitometry with Pulmozyme® as a control.

### SDS-PAGE with coomassie or silver staining

Cell extracts, culture SN, eluates, or purified proteins were analyzed by SDS-polyacrylamide gel-electrophoresis [57]. Samples were denaturized for 5 minutes at 95°C in SDS-sample buffer (62.5 mM Tris HCL, pH 6.8, 2% (w/v) SDS, 10.0% (v/v) glycerol, 5% (v/v) β-mercaptoethanol, 0.001% (w/v) bromophenol blue) [84]. After electrophoresis, gels were stained with Coomassie brilliant blue G-250 [57] or with silver nitrate. Routinely 250 µL concentrated or 25 µL pure SN were used for investigation by Coomassie- or silver gels, respectively. For silver staining, the gels were incubated for 20 minutes in each 100 mL of distilled water, fixation solution (50% (v/v) ethanol, 10% (v/v) acetic acid), oxidation solution (30% (v/v) ethanol, 0.5 M sodium acetate, 1.27 mM sodium thiosulfate, 0.8% (v/v) glutaraldehyde), distilled water, and silver solution (5.9 mM silver nitrate in 0.01% (v/v) formaldehyde). Gels were developed in a solution of 236 mM sodium carbonate, 0.02% (v/v) formaldehyde, and 0.5 mM thimerosal. Silver reduction was stopped with 50 mM EDTA in 5% (v/v) acetic acid.

### Denaturing PAGE zymography

Denaturing PAGE zymography (DPZ) was performed as described [57] using gels with a 10% (v/v) resolving part containing 100 µg/mL calf thymus DNA. Samples were denatured like for SDS-PAGE. For detection of mDNase1 usually 0.2–0.5 µL culture SN were investigated compared to 7.5–11.25 µL for hDNASE1. After washing twice at 50°C (removal of SDS and pH adjustment), the gels were incubated for 16 hours at 26–37 °C (depending on the signal strength) in 10 mM Tris/HCl, pH 7.8–8.0 containing each 2 mM CaCl_2_ and MnCl_2_ for nuclease reaction, i.e., at conditions supporting similar specific activities of human and mDNase1. Gels were stained with ethidium bromide.

### Native PAGE zymography

For native PAGE zymography (NPZ) 7.5% (v/v) polyacrylamide gels without SDS containing 20 µg/mL calf thymus DNA were prepared. The gel- and running buffer consisted of 25 mM Tris/HCl, pH 8.3–8.6 and 192 mM glycine. Protein samples were dissolved in running buffer containing 50% (v/v) glycerol and 0.001% (w/v) bromophenol blue. Electrophoresis was done for 1.5 hours at 100 V. Afterwards, the gels were washed for 30 minutes in 25 mM Tris/HCl, pH 7.8–8.0 at 37 °C (pH adjustment). Nuclease reaction occurred for ~ 20 minutes at 37 °C in incubation buffer identical to that used for DPZ prior to staining the gels.

### Gel Imaging

Images of agarose or acrylamide gels were captured using the ChemiDoc™ XRS + System with the ImageLab 5.0 software and evaluated densitometrically when appropriate.

### Diethylaminoethyl-cellulose anion-exchange chromatography

For purification of DNase1 from SN, DEAE-cellulose chromatography was employed as described [57,85]. Thus, SN was concentrated using 10 K centrifugal filters and dialyzed with chromatography (C) buffer (25 mM MES, pH 6) using 5 K centrifugal filters. Then, it was passed through a glass column, loaded with activated and equilibrated DEAE-cellulose DE53 at a flow rate of 1 mL/minute. After washing with C-buffer, elution performed with C-buffer containing 50–500 mM NaCl. Finally, eluates E150 and E200 were employed in gel-filtration or hydroxyapatite chromatography.

### Gel-filtration

For gel-filtration, 80 mL swollen Bio-Gel P-100 were loaded into a glass column and equilibrated with filtration buffer (5 mM sodium phosphate, pH 6.8, 300 mM NaCl, 1 mM NaN_3_). Concentrated eluates E150 and E200 of the DEAE- for murine or E50 of the HAP chromatography for hDNASE1 were loaded on the gel bed and eluted with filtration buffer as described [57]. Fractions of 1 mL were collected and analyzed by SDS-PAGE with silver staining and by HCA to identify those containing DNase1. These fractions were pooled and purified by a final HAP chromatography.

### Hydroxyapatite chromatography

About 750 µL Bio-Gel HT hydroxyapatite (HAP) were loaded into a glass column and equilibrated with 5 mL C-buffer (5 mM sodium phosphate, pH 6.8, 300 mM NaCl). For hDNASE1, HAP chromatography was done before and for both orthologues after gel-filtration according to John and Schmidt [86]. Before gel-filtration the DEAE E150 and E200 elution fractions were supplemented with sodium phosphate, pH 6.8 and NaCl to 5 and 300 mM, respectively, and loaded onto the column. The column was washed with 5 mL C-buffer and elution was performed with C-buffer containing 10–250 mM sodium phosphate, pH 6.8. Fractions containing DNase1 were employed in gel-filtration. For HAP chromatography after gel-filtration, DNase1 containing fractions were directly loaded onto the HAP column. After washing with 5 mL C-buffer, elution was performed as described above. Eluted fractions containing DNase1 were concentrated and dialyzed against storage buffer (25 mM MES, pH 6, 150 mM NaCl, 2 mM CaCl_2,_ 1 mM NaN_3_) using 3 K centrifugal filters.

### Hyperchromicity assay

Nuclease activity was measured using the hyperchromicity assay (HCA) according to Kunitz 1950 [7]. The reaction solution (1 mL) consisted of 50 µg/mL calf thymus DNA dissolved in 10 mM buffer with 0.1 mM CaCl_2_, and either 1 mM MgCl_2_ or MnCl_2_. The pH was adjusted using acetate- (pH 5.0–5.5), MES- (pH 6.0–6.5), or Tris-buffer (pH 7.0–9.0). Absorbance at 260 nm was recorded for 30 seconds at room temperature in a quartz cuvette with a 1 cm path length using a Beckman DJ640 spectrophotometer. One unit is defined by an increase in absorbance of 0.001 per minute. Samples were measured at least in duplicate. Inhibition of DNase1 with skeletal muscle α-actin was determined after pre-incubation with skeletal muscle G-actin in 5 mM HEPES, pH 7.4 with 0.2 mM ATP, and 0.1 mM CaCl_2_ for 20 minutes at room temperature before measuring the activity at pH 8 in the presence of 0.1 mM CaCl_2_ and 1 mM MnCl_2_.

### De-N-glycosylation

For de-glycosylation, DNase1 was denatured either with glycoprotein denaturing buffer containing 0.5% (w/v) SDS and 40 mM DTT or with DTT only for 10 minutes at 95 °C. Subsequently, 250 units RemoveiT® PNGase F in 2x GlycoBuffer 2 were added to the samples denatured with only DTT. This treatment only led to a partial de-glycosylation, whereas adding PNGase F in 2x GlycoBuffer 2 containing 1% (v/v) NP-40 to the samples denatured in the presence of SDS and DTT led to full de-glycosylation. After de-N-glycosylation for 1 hour at 37 °C, SDS-sample buffer was added, PNGase F was denatured at 95 °C, and the samples were analyzed by SDS-PAGE with silver staining.

### Statistical analysis

Data determined by HCA or densitometry of gels were evaluated by an unpaired student`s T-test as indicated. Coomassie-, silver-, DPZ- or NPZ gels of SN and cell extracts were always evaluated semi-quantitatively by comparison of signals derived from of up to three yeast clones or of samples collected within a consecutive test series. Conclusions were only drawn from samples compared on the same gel.

## Results

### Comparative expression of αMF-DNase1

**Fig 2:**
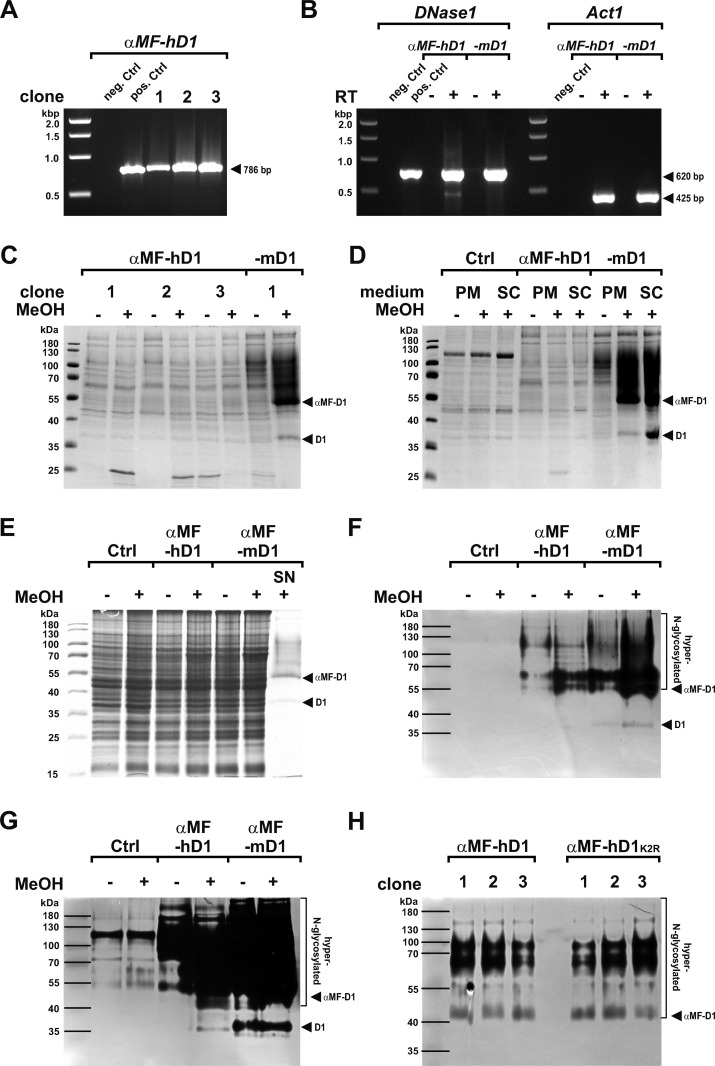
Comparative expression ofαMF-DNase1. (A) Proof of the αMF-hDNASE1 (αMF-hD1) transgene in clones of *PichiaPink™ pastoris* strain 4 by PCR (neg. Ctrl: water, pos. Ctrl: vector pPinkα-HC/αMF-hDNASE1). (B) Detection of *DNase1* and *Act1* mRNA in each one clone for αMF-hDNASE1 and -mDNase1 (αMF-mD1) by RT-PCR. Controls (Ctrl) without reverse transcriptase (-RT) indicate lack of genomic DNA contamination. (C) Pilot expression of αMF-hDNASE1 compared to -mDNase1 with no clear detection of premature (αMF-D1) or mature (D1) hDNASE1 in the SN as evaluated by Coomassie gel analysis. Samples without MeOH (-) served as negative Ctrl. (D) Variation of the expression medium between peptone (PM) and synthetic complete adenine drop-out medium (SC) did not improve expression of αMF-hDNASE1 in exemplary clone 2 as shown by Coomassie gel analysis. The non-inducible protein of ~ 37 kDa in the SN of αMF-hDNASE1 cells also occurs in the SN of non-transformed Ctrl cells. Maturation of αMF-mDNase1 is improved using SC medium. (E) Lack of detectable intracellular expression products in cell extracts by Coomassie gel analysis of protein isolated from each 1 mg cells. (F, G) Verification of premature hyper-N-glycosylated αMF- and mature mDNase1 as well as hDNASE1 in SN by DPZ: (F) 2 µL WT Ctrl and αMF-hDNASE1 vs. 0.2 µL of -mDNase1, (G) 5 vs. 0.5 µL. (H) Adaption of the N-terminus of mature human to that of mDNase1 by a K2R mutation with no improvement of αMF-hDNASE1 expression as shown by DPZ of SN. Marker: 1kb DNA Ladder (A, B) and PageRuler™ Prestained Protein Ladder (C-H). Data shown for single exemplary experiments with one to three clones as indicated.

Recently we expressed mDNase1 using the entire αMF-SP in *P. pastoris* and found an inefficient pro-peptide cleavage. Full maturation could only be achieved by employing enzymes co-occurring in the expression culture supernatant (SN) [57]. Analogous to mDNase1, we now cloned an expression vector for αMF-hDNASE1 (pPinkα-HC/αMF-hDNASE1) and selected transformed clones of *PichiaPink*^*TM*^ *pastoris* strain 4. Stable transgene integration and induced transcription were proven by PCR and RT-PCR, respectively (Fig 2A and B).

Expression was done according to the so far optimized conditions for αMF-mDNase1 [57]. In short, 0.25x BMGY was used for growth and PM medium with 0.5% (w/v) peptone and 0.5% (v/v) MeOH for expression. Growth cultures were grown to OD_600_ =  6 at 28 °C, washed with PBS to remove transcription inhibiting glycerol [57], and low scale expression cultures with OD_600_ =  60 were incubated for 24 hours at 28 °C. As a result, we found by SDS-PAGE that in contrast to αMF-mDNase1, no premature αMF- nor mature hDNASE1 were detectable in the SN. Only a faint but not inducible protein of ~ 37 kDa occurred similar to di-N-glycosylated mature mDNase1 in the SN of αMF-hDNASE1 expressing cells (Fig 2C). However, it was also detectable in WT control making it doubtful to represent mature hDNASE1 (Fig 2D). Lowering nutrients by using SC-Ade expression medium improved maturation only of mDNase1 (Fig 2D) [57].

Since mRNA for αMF-hDNASE1 was verified (Fig 2B), we analyzed whether DNase1 was detectable intracellularly. However, no induced functional or altered DNase1 was found by SDS-PAGE in extracts of transgenic cells for each orthologue (Fig 2E). To increase detection, we investigated the expression SN with DPZ, i.e., by SDS-PAGE with gels containing DNA. This method is more sensitive and has the advantage that nuclease activity can be assigned to distinct protein bands. Interestingly, αMF-hDNASE1 expression was verified with the same protein pattern as -mDNase1 (Fig 2F and G). However, the expression level was ten times lower compared to non-induced αMF-mDNase1 as deduced from the volumes of SN analyzed (Fig 2F). In addition, we used the photometric hyperchromicity assay (HCA) to detect nuclease activity. In contrast to αMF-mDNase1, which was detectable in small volumes of SN, we were unable to detect -hDNASE1 (routinely 20 µL SN were analyzed). Thus, its expression is in the range of background detection, i.e., below 25 ng/mL Pulmozyme®, which served as control. Therefore, we concentrated larger amounts of SN and found that compared to αMF-mDNase1, the induced expression of -hDNASE1 was 70–80 times lower (14.4 ±  2.5 vs. 0.2 ±  0.04 kU/mL, p <  0.001) measured at conditions ensuring similar specific activities for both (Ca^2 + ^/Mn^2 +^ at pH 8).

Next, we tried to rule out that the different expression efficiencies resulted from an ineffective cleavage of the αMF pro-peptide at the N-terminus of DNase1 by Kex2, which might lead to negative feedback on expression. Therefore, we adapted the N-terminal sequence of mature human to that of mDNase1 by introducing a K2R mutation (hDNASE1^K2R^). Unfortunately, this had no effect on the expression level nor the maturation of αMF-hDNASE1 (Fig 2H).

### Comparative expression of preαMF-DNase1

**Fig 3:**
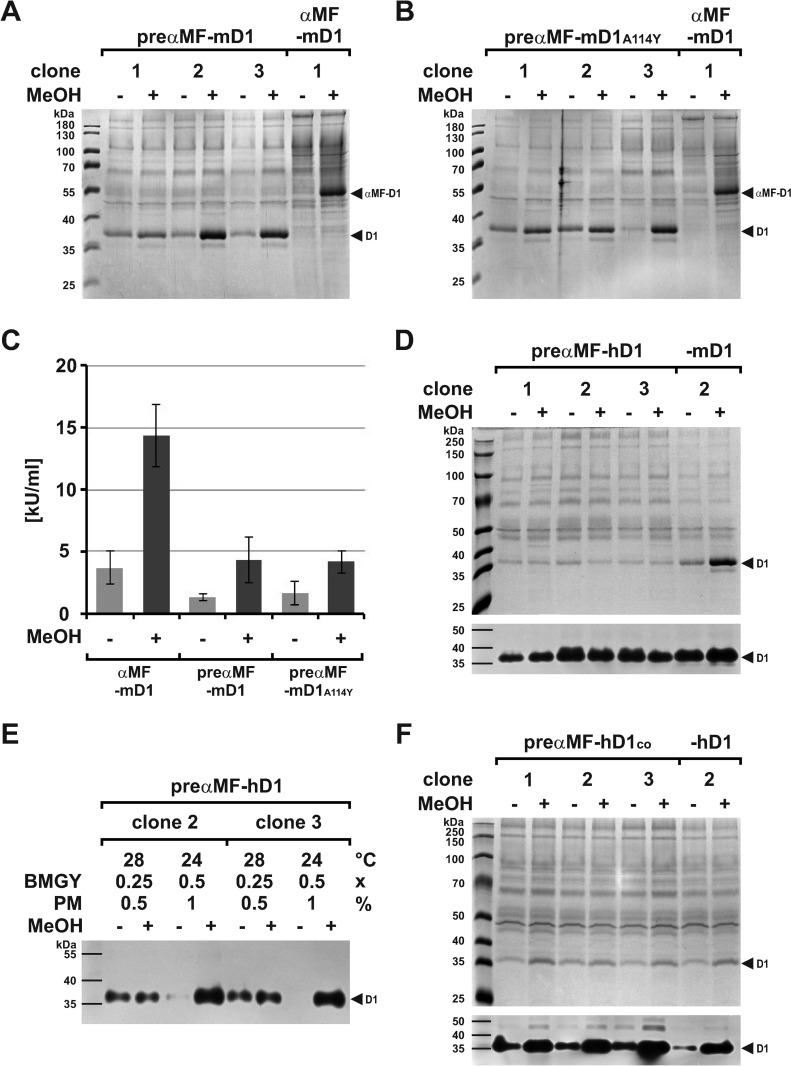
Comparative expression of pre **αMF-DNase1.** (A, B) Pilot expression of preαMF-mDNase1 and the actin-resistant mutant -mDNase1^A114Y^ compared to αMF-mDNase1 with secretion of only mature mDNase1 (~37 kDa) due to deletion of the αMF pro-peptide as deduced from Coomassie gel analysis of SN. (C) Mean DNase1 activity in the SN of the clones shown in (A) and (B) as determined by HCA shows a threefold lower level of preαMF-mDNase1 and -mDNase1^A114Y^ compared to αMF-mDNase1 (each **p** <  0.001). (D) Pilot expression of preαMF-hDNASE1 compared to -mDNase1: Expression of preαMF-hDNASE1 with low induction by MeOH, but detectability by DPZ (11.25 µL SN with hD1 vs. 0.75 µL SN with mD1) as presented below the Coomassie gel. (E) Verification of the optimization presented in S2 Fig by adapting nutrients and cultivation temperature during growth and expression as shown by DPZ of SN. Clear inducibility and a slightly higher expression of preαMF-hDNASE1 under optimized conditions. (F) Pilot expression of preαMF-hDNASE1^co^ compared to -hDNASE1 under optimized nutrient conditions at 24 °C with a slightly higher expression due to codon optimization (co) as evaluated by Coomassie gel analysis of SN and DPZ below. Marker: PageRuler™ Prestained Protein Ladder (A–B) and Prestained protein marker PAN-Biotech™ (D–F). Data shown for single exemplary experiments with one to three clones as indicated (numbers of the αMF-DNase1 clones refer to Fig 2).

Inefficient cleavage of the αMF pro-peptide is a limitation for DNase1 expression with the *PichiaPink™* system. To overcome this, we cloned an expression vector with only the pre-peptide fused to the N-terminus of mature mDNase1 (pPinkHC/preαMF-mDNase1), transformed strain 4, and investigated clones as described above. By SDS-PAGE we found that pro-peptide deletion led to secretion of solely mature mDNase1 (Fig 3A).

However, HCA revealed that compared to αMF-mDNase1, preαMF-mDNase1 expression decreased threefold (Fig 3C: 14.4 ±  2.5 vs. 4.3 ±  1.8 kU/mL, p <  0.001), probably due to the loss of two described pro-peptide functions [76,77]. A delayed rER import for example might induce nucleolytic cytotoxicity due to cytoplasmic accumulation and diffusion of mDNase1 into the nucleus [17], though its inhibition by G-actin may fulfill a safety mechanism [80]. Therefore, we expressed mDNase1^A114Y^ harboring a mutation eliminating actin-binding [49,80]. This should lead to increased cytotoxicity and decreased expression. However, preαMF-mDNase1^A114Y^ was expressed equally well as WT mDNase1 (Fig 3B and C: 4.3 ±  1.8 vs. 4.2 ±  0.9 kU/mL). Therefore, we conclude that pro-peptide deletion impaired rER import of DNase1 without inducing cytotoxicity. Additionally, the transfer and export of DNase1 through and from the secretory compartment might be affected.

Analogous to mDNase1, we expressed hDNASE1 with only the αMF pre-peptide. Unfortunately, its expression remained very low and was not clearly detectable by SDS-PAGE. Although a faint protein band of ~ 37 kDa co-migrating with mDNase1 was visible in the hDNASE1 samples again, this protein was not inducible by MeOH (Fig 3D). However, with DPZ preαMF-hDNASE1 expression was verified (Fig 3D). Evaluation of the expression using concentrated SN by HCA revealed a ~ 25 times lower level for human compared to preαMF-mDNase1 (0.2 ±  0.04 vs. 4.7 ±  0.1 kU/mL, p <  0.001). In comparison to the difference of 70–80 times when using the entire αMF-SP, pro-peptide deletion led to a threefold approximation in expression, which however correlated with an identical reduction of mDNase1 expression (Fig 3C). Therefore, it can be assumed that hDNASE1 expression was if at all only marginally affected. Its expression remained on the same low level without support by the αMF pro-peptide. The advantage so far is the production of pure mature DNase1.

### Optimization of preαMF-DNase1 expression

#### Influence of nutrients and cultivation temperature.

To increase expression of especially preαMF-hDNASE1, we evaluated the composition of the growth and expression media and lowered the cultivation temperature from 28 to 24 °C. Increasing nutrients in the growth medium above 0.25x BMGY improved induction and reduced background expression at both temperatures using 0.5x BMGY (S2 Fig). An additional increase of peptone above 0.5% (w/v) in the expression medium had only a minor effect, but 1% PM appeared to be optimal (S2 Fig). Since the DPZ is difficult to standardize, samples can only be evaluated semi-quantitatively on the same gel. Therefore, we compared the influence of nutrients and temperature on a combined gel and found that expression saturated at media composition of 0.5x BMGY and 1% PM at 28 °C (S2 Fig). Nevertheless, optimization of nutrients was also detectable at 24 °C as shown for two clones expressing preαMF-hDNASE1 (Fig 3E). Compared to previous results, expression of hDNASE1 saturated at a higher nutrient supply than mDNase1, where 0.25–0.5x BMGY and 0.5% PM was optimal at 28 °C [57]. For the following experiments, we subsequently used 0.5x BMGY for growth and 1% PM for expression since it guaranteed saturated conditions for both orthologues. Pilot expressions always started at 24 °C and for evaluation of further improvement we switched to 28 °C to reveal the overall effect.

#### Influence of the codon usage and transgene dose.

In addition to nutrients and cultivation temperature, expression limitations might be a different codon usage between host and graft and inhibitory secondary mRNA structures [87–90]. Therefore, codon optimized preαMF-hDNASE1^co^ was expressed (Fig 3F). Adaption encompassed codons, which occur to less than 25% in *P. pastoris*, i.e., 20 of 261 codons (7.7%) of the *hDNASE1* mRNA were exchanged to the most commons (S3 File) [91]*.* Thereby, the codon adaptive index raised from 0.58 to 0.67, which resembles that of mDNase1 (0.65). In addition, the predicted secondary mRNA structure changed leading to an approximation of the free energy between the thermodynamic ensemble of the mRNAs (Vienna RNA Web Services, Institute for Theoretical Chemistry). However, codon optimization and the altered mRNA structure had only a slightly enhancing effect on hDNASE1 expression (Fig 3F).

**Fig 4:**
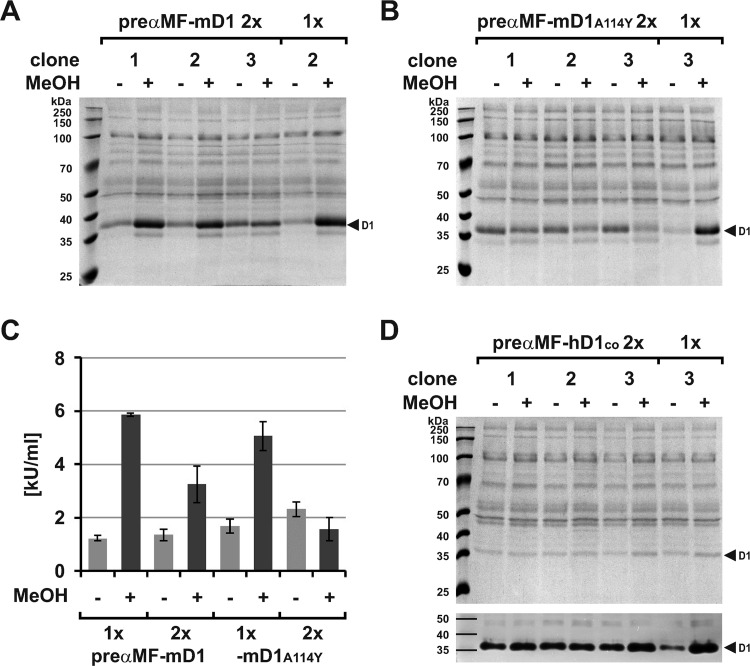
Influence of the transgene dose on pre **αMF-DNase1 expression.** (A, B) Pilot expression of preαMF-mDNase1 and -mDNase1^A114Y^ employing two (2x) compared to one (1x) expression cassette as evaluated by Coomassie gel analysis of SN. (C) Mean mDNase1 activity in the SN of the clones shown in (A) and (B) as determined by HCA showing lower induced expression using two cassettes (mDNase1, **p** =  0.0014 and mDNase1^A114Y^, **p** <  0.001). Background expression slightly increased for mDNase1^A114Y^ (p =  0.0062). (D) Comparable results obtained for preαMF-hDNASE1 as evaluated by Coomassie gel analysis of SN and DPZ below. Marker: Prestained protein marker PAN-Biotech™. Data shown for single exemplary experiments with one to three clones as indicated (numbers of the clones with a single expression cassette refer to Fig 3).

A further approach to enhance expression can be an increased transgene number [92]. To analyze this dose effect, we cloned a second expression cassette into the pPinkHC/preαMF-DNase1 vectors and transformed them. Surprisingly, the induced yields of preαMF-mDNase1 and its actin-mutant decreased by ~ 46% (Fig 4C: 5.9 ±  0.1 vs. 3.3 ±  0.7 kU/mL, p = 0.0014) and ~ 69% (Fig 4C: 5.1 ±  0.5 vs. 1.6 ±  0.4 kU/mL, p <  0.001) compared to a single cassette, whereas background expression slightly increased for the actin-mutant (Fig 4A–C).

The experiment for preαMF-hDNASE1^co^ led to comparable results (Fig 4D). Thus, a positive gene dose effect was not detectable at induced conditions probably due to a limitation at the translational and/or post-translational level for both orthologues. Apparently, too much transcript induced negative feedback, though cytotoxicity by higher amounts of DNase1 is still conceivable.

#### Influence of nucleolytic cytotoxicity.

Although cytotoxicity by actin-resistant mDNase1 was not verified, its more reduced expression compared to the WT isoform when using two expression cassettes might indicate that this possibility still exists. To finally rule it out, we expressed DNase1 harboring a D168S loss-of-function mutation in the catalytic center [47]. Interestingly, no positive effect was observed. For preαMF-mDNase1^D168S^ the expression level was reduced compared to WT mDNase1, whereas there was no detectable effect for preαMF-hDNASE1^D168S^ (S3 Fig). The DPZ demonstrated lack of activity for both mutated orthologues. Thus, we found no indication of DNase1 induced cytotoxicity as a limitation of its expression.

#### Influence of intra- and extracellular factors on DNase1 protein stability.

Heterologous secretory protein expression harbors the risk, that the proteins are affected by host proteases or by the pH of the culture media used [93,94]. Routinely we employed *PichiaPink*^*TM*^ *pastoris* strain 4 lacking vacuolar aspartyl protease proteinase A, carboxypeptidase Y, and serine protease proteinase B [95]. However, other proteases might act disruptive. To evaluate these restrictions, preαMF-mDNase1 and -hDNASE1^co^ were expressed in the presence of varying pH, protease inhibitors and in *PichiaPink*^*TM*^ *pastoris* strains lacking the above-mentioned proteases in different combinations. For both orthologues, optimal expression occurred at pH 6–7 without proteolysis or degradation (S4 Fig). To rule out destabilizing effects on especially hDNASE1, 300 µg Pulmozyme® were added to WT cells, mock-transfected with vector pPinkHC. After 24 hours, Pulmozyme® was purified from the expression SN as described (DEAE chromatography and P-100 gel-filtration) [57]. We retrieved 130 µg with a reduced specific activity of ~ 32% (S4 Fig: 6.8 ±  0.3 vs. 10.0 ±  0.3 kU/nmol, p <  0.001) compared to the origin when applying the overall protein amount of the isolate as determined by the Bradford assay. Nevertheless, correcting the amount of purified Pulmozyme® in the isolate by an alignment with the specific activity of the origin and by counter calculating the alignment by silver gel analysis of each 1 µg de-N-glycosylated protein proved that the purified Pulmozyme® was nearly unaffected (S4 Fig). Since the protein amount of the isolate did not match with that of purified Pulmozyme®, the purification procedure is not specific enough. The loss of Pulmozyme® is most likely caused by adsorption to cell surfaces or debris, metabolization, and/or losses during the different steps of purification.

#### Influence of the methanol supply.

**Fig 5:**
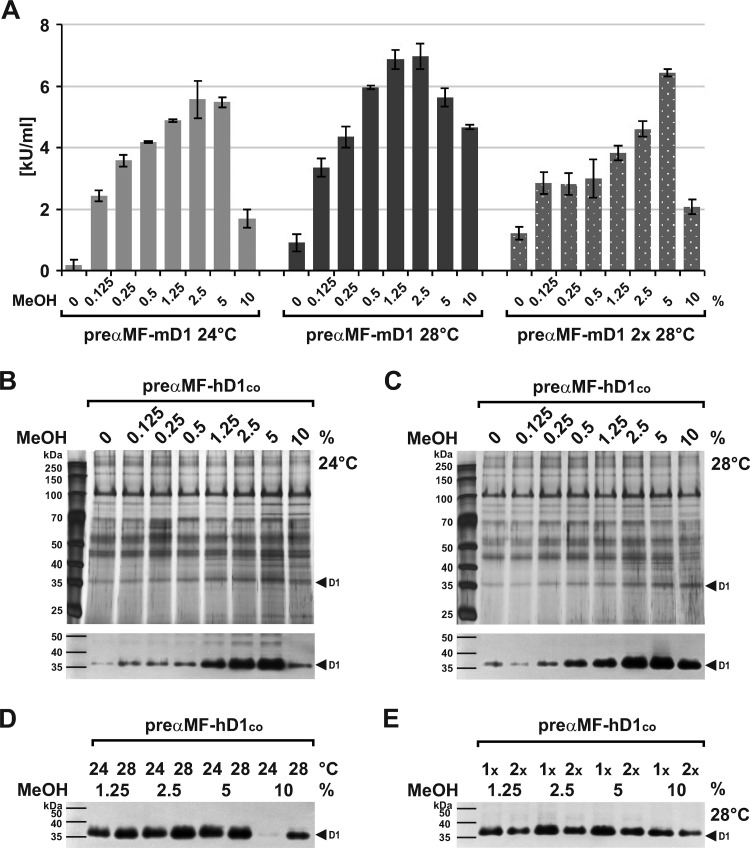
Influence of the methanol supply on DNase1 expression. (A) Raising MeOH above 0.5% (v/v) in the expression medium increases preαMF-mDNase1 production with an optimum at 5% MeOH and 24 °C (p =  0.0059) and even more at 2.5% and 28 °C (p =  0.0441) as determined by HCA of SN. Employing two expression cassettes (2x) led to a delayed rise. (B, C) Identical results obtained for preαMF-hDNASE1^co^ as illustrated by silver gel analysis of SN combined with DPZ below. (D) Expression of preαMF-hDNASE1^co^ at optimal 5% MeOH and 24 °C is slightly lower to 2.5% MeOH and 28 °C as evaluated by DPZ. (E) Comparable to preαMF-mDNase1, two preαMF-hDNASE1^co^ transgenes (2x) delay and decrease expression as shown by DPZ. Marker: Prestained protein marker PAN-Biotech™. Data shown for single exemplary experiments using clone 2 and 1 for preαMF-mDNase1 1x and 2x as well as clone 3 and 1 for preαMF-hDNASE1^co^ 1x and 2x presented in Fig 3 and 4.

Important for protein expression controlled by the AOX1 promoter is the MeOH supply [59]. Therefore, we tested different amounts and found the so far used 0.5% (v/v) proposed by the supplier to be not optimal [78]. A rise to 5% MeOH increased preαMF-mDNase1 expression by ~ 30% at 24 °C (Fig 5A: 4.2 ±  0.03 vs. 5.5 ±  0.2 kU/mL, p =  0.0059). Analogous to the results obtained before, this was further elevated to ~ 67% (4.2 ±  0.03 vs. 7.0 ±  0.4 kU/mL, p =  0.0027) by raising the cultivation temperature to 28 °C already using 2.5% MeOH. Interestingly, a delayed rise of expression was observed using two transgene cassettes under increasing MeOH (Fig 5A), which cannot be explained by a linear heightening of transcription compared to a single cassette, i.e., by a gene dose effect.

It can be assumed that MeOH beside transcription also affects the energy metabolism of the Mut^ +^ cells in the absence of further carbon sources and that a high amount of transcript without sufficient energy acts inefficient. Therefore, we assume that expression is actively downregulated at insufficient energy for post-transcriptional processes. Fortunately, also preαMF-hDNASE1^co^ could be enhanced by an increased MeOH supply and a raised cultivation temperature (Fig 5B–D). Unfortunately, it also showed an inverted gene dose effect, which in contrast to preαMF-mDNase1 could not be entirely compensated by increased MeOH (Fig 5E).

### Comparative expression of preOst1-DNase1

Several restrictions for protein expression in *P. pastoris* are described [64] and our results reveal that the production maxima for DNase1 do not result from a limited transcription. Thus, further bottlenecks like rER import, protein folding, transfer, and secretion have to be considered. As described, the αMF-SP induces post- [75] whereas the Ost1-SP induces co-translational rER import [81] corresponding to the physiological DNase1 transport in mammalian cells as proven by GFP-tagging [17].

**Fig 6:**
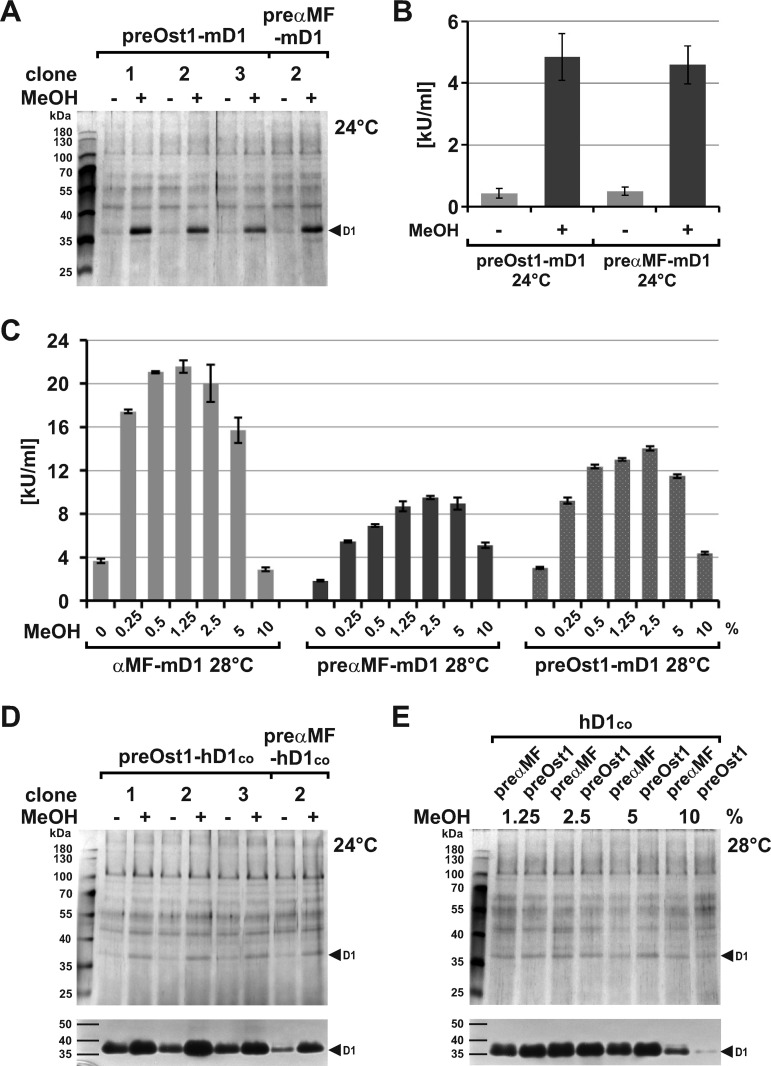
Comparative expression of preOst1-DNase1. (A) Pilot expression of preOst1- compared to preαMF-mDNase1 with similar results at 24 °C using suboptimal 0.5% (v/v) MeOH as evaluated by silver gel analysis of SN. (B) Mean DNase1 activity in the SN of the clones shown in (A) as determined by HCA. (C) Raising the cultivation temperature to 28 °C and MeOH to 2.5% enhanced preαMF- and even more preOst1-mDNase1 (clone 1) expression (p <  0.001) proving that co- is superior to post-translational rER import at 28 °C. Compared to αMF-mDNase1, the threefold reduction due to the pro-peptide deletion at 0.5% MeOH was partially compensated by expressing preαMF-mDNase1 with 2.5% MeOH (~13%, **p** <  0.001) and additionally by a switch to co-translational import using preOst1-mDNase1 at 2.5% MeOH (~22%, **p** <  0.001) as determined by HCA. (D) Pilot expression of preOst1- compared to preαMF-hDNASE1^co^ reveals that co-translational import is already superior at 24 °C using 0.5% MeOH as shown by silver gel analysis of SN and DPZ below. (E) Comparison of preOst1- (clone 2) with preαMF-hDNASE1^co^ at 28 °C combined with increased MeOH reveals that all positive factors (temperature, MeOH, and SP) do not fully act additively but led to a saturated expression as shown by silver gel analysis and DPZ below. Marker: PageRuler™ Prestained Protein Ladder. Data shown for single exemplary experiments employing one to three clones as indicated (numbers of the preαMF-DNase1 clones refer to Fig 3).

To evaluate rER import we changed to the Ost1-SP and transformed strain 4 with pPinkHC/preOst1-mDNase1 and -hDNASE1^co^. However, pilot expression of preOst1- compared to preαMF-mDNase1 using 0.5% (v/v) MeOH and optimized nutrients had no effect at 24 °C (Fig 6A and B).

Comparable to the results described above, raising MeOH to 2.5% and the cultivation temperature to 28 °C improved preαMF-mDNase1 expression twofold (Fig 6B and C: 4.6 ±  0.6 vs. 9.5 ±  0.1 kU/mL, p <  0.001). However, when using the Ost1-SP a threefold increase (Fig 6B and C: 4.8 ±  0.4 vs. 14.0 ±  0.2 kU/mL, p <  0.001) was achieved, indicating that post-translational rER import of mDNase1 is a limiting factor with rising temperature. When comparing αMF- with preαMF-mDNase1 expression, we found that the threefold reduction due to the pro-peptide deletion described above (Fig 3C) also remained under optimized nutrient conditions using 0.5% MeOH at 28 °C (Fig 6C: 21.1 ±  0.1 vs. 6.9 ±  0.1 kU/mL, p <  0.001). In contrast to αMF-mDNase1 expression, which saturated with optimized nutrients despite an additional raise of MeOH (Fig 6C: 21.1 ±  0.1 with 0.5% and 21.6 ±  0.6 kU/mL with 1.25% MeOH compared to Fig 3C: 14.4 ±  0.25 kU/mL with 0.5% MeOH, p =  0.0037), preαMF-mDNase1 expression further increased so that ~ 13% of the difference to αMF-mDNase1 expression were compensated (p <  0.001). Thus, increased MeOH partially substitutes the pro-peptide deletion. Since MeOH did not induce a positive gene dose effect, it most likely caused an improved post-translational processing by providing a higher energy supply. Changing post- to co-translational import by employing the Ost1-SP additionally compensated ~ 22% when using 2.5% MeOH (p <  0.001), i.e., the threefold reduction due to the pro-peptide deletion could be halved (Fig 6C: 21.6 ±  0.6 vs. 14.0 ±  0.2 kU/mL, p <  0.001) leaving a difference of ~ 35%.

When comparing the results for preαMF- and preOst1-hDNASE1^co^ with those of mDNase1, we found that co-translational import was already superior at 24 °C (Fig 6D) implicating that post-translational transfer of hDNASE1 is already delayed at lower temperatures. When increasing the cultivation temperature to 28 °C and MeOH to 2.5%, hDNASE1 expression with both SP equalized at a maximum revealing that the rER import is not the sole limitation for its expression (Fig 6E). Optimizing the limiting aspects so far resulted in expression maxima, but especially for hDNASE1 it was difficult to quantify the contribution of each individual factor. To compensate the residual expression difference caused by the αMF pro-peptide deletion, which most likely resulted from a disturbed protein transfer from the rER to the GA, we again evaluated a gene dose effect. Comparable to preαMF-DNase1 2x, also the expression of preOst1-mDNase1 2x and -hDNASE1^co^ 2x had no positive effect suggesting that the limitation of expression is not only due to post-translational import, but also to processes within the rER (S5 Fig). Thus, for both orthologues the expression maximum is already achieved with a single expression cassette.

#### Kinetics of preOst1-DNase1 expression.

**Fig 7:**
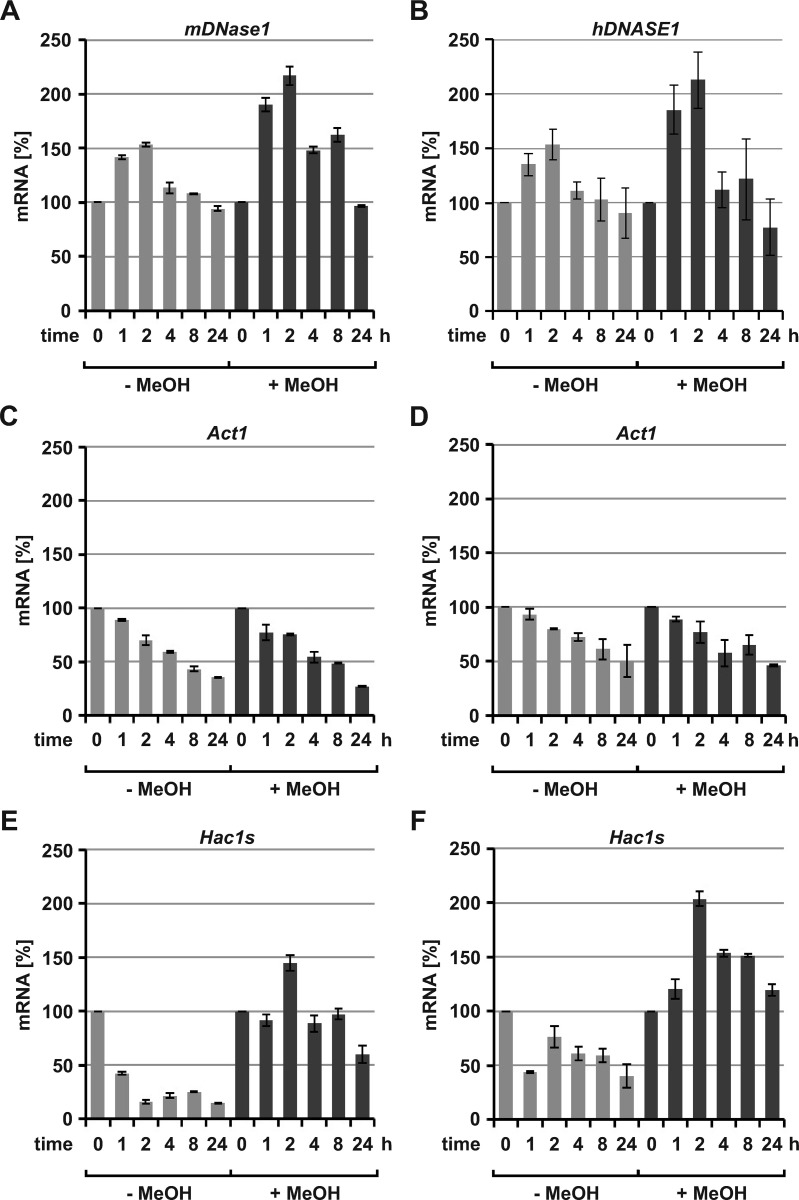
Kinetics of preOst1-DNase1 expression at the transcriptional level. Analysis by RT-PCR and densitometry after gel-electrophoresis. Results shown for preOst1-mDNase1 (left column: A, C and E) and -hDNASE1^co^ (right column: B, D and F). Expression performed with 1% (w/v) PM and 2.5% (v/v) MeOH at 28 °C for 24 hours. (A, B) Transcription of *DNase1* under non-induced (- MeOH) and induced (+) conditions shows similar kinetics for both orthologues: Induced transcription decreased rapidly two hours after its maximum (mDNase1 **p** =  0.0082, hDNASE1 **p** <  0.001) which is more pronounced for *preOst1-hDNASE1*^*co*^ (148.5 ±  2.8 vs. 112.2 ±  16.3%, **p** =  0.031). (C, D) Transcription of *Act1* (actin) with identical declining kinetics over 24 hours during induced (each **p** <  0.001) and non-induced expression (mDNase1 **p** <  0.001, hDNASE1 **p** =  0.0421) indicating a general loss of cell viability and/or growth arrest. (E, F) Evaluation of an UPR by detection of the *Hac1s* mRNA with a stronger (2 hours: 203.9 ±  6.8 vs. 144.7 ±  7.0%, **p** =  0.0133) and prolonged (24 hours: 119.1 ±  5.4 vs. 59.9 ±  8.2%, **p** =  0.0135) induction during preOst1-hDNASE1^co^ compared to -mDNase1 expression. Washing cells with PBS prior to expression also appeared to activate an UPR: non-induced Ctrl, 0 - 1 hour (each **p** <  0.001). After transfer into expression medium, *Hac1s* dropped and was reactivated only in non-induced cells expressing preOst1-hDNASE1^co^ (p =  0.0437). Data shown for one exemplary experiment employing clone 1 for each preOst1-mDNase1 and -hDNASE1^co^ (Fig 6). Mean of a duplicate RT-reaction with at least two to three RT-PCRs per reaction and sample.

Next, we tried to decipher the limitations related to post-translational processes within the rER which might explain the expression difference between both DNase1 orthologues and compared the kinetics of transcription to that of translation. Therefore, gene expression at optimal nutrient, temperature, and MeOH conditions for preOst1-mDNase1 and -hDNASE1^co^ was investigated over 24 hours. The induction phase of transcription, i.e., the time to reach the highest mRNA level, lasted only two hours (Fig 7A and B).

Correlating with its reduced expression, transcription of *preOst1-hDNASE1*^*co*^ decreased to a higher extend than that of *-mDNase1*. Identical kinetics were observed for non-induced controls showing only half maximal induced expression. It appeared that the cells after transfer into carbon source free medium metabolized residual glycerol resulting in a disinhibition of the AOX1 promoter.

A biphasic kinetic comparable to *DNase1* transcription could not be observed for actin. Surprisingly its transcription dropped with ongoing cultivation hinting to a stationary phase with growth arrest and/or loss of cell viability, which occurred independently of the DNase1 orthologue expressed (Fig 7C and D).

**Fig 8:**
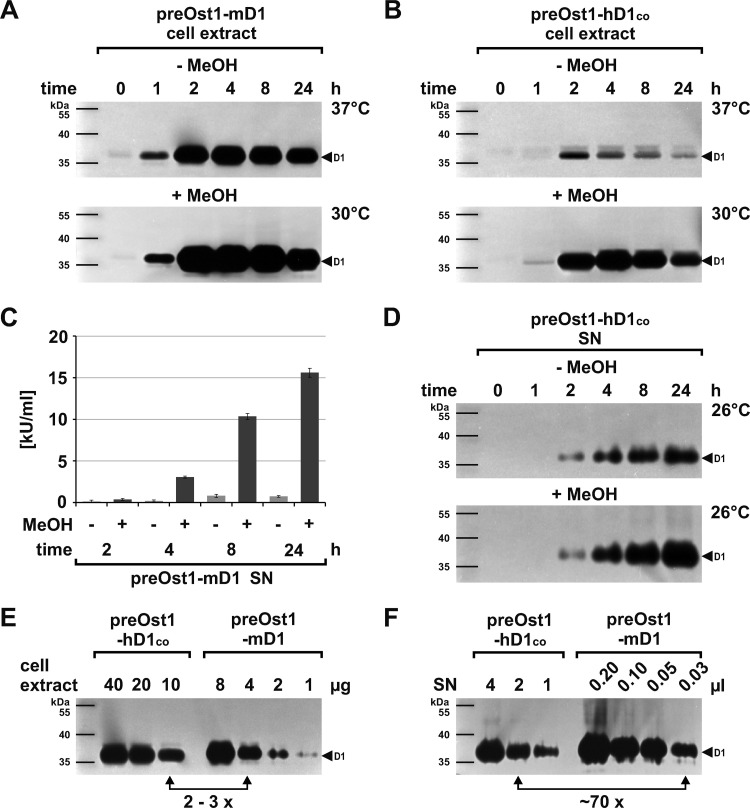
Kinetics of preOst1-DNase1 expression at the translational level. Time dependent appearance of (A) murine and (B) hDNASE1 in cell extracts with a comparable translation kinetic as evaluated by DPZ: 20 vs. 40 µg extract for induced and non-induced preOst1-mDNase1 and -hDNASE1^co^ expression, respectively. Corresponding kinetics with a delay of one hour were detectable for the occurrence of DNase1 in the SN as shown for (C) mDNase1 determined by HCA and (D) hDNASE1 by DPZ. (E, F) Semi-quantitative analysis of the different expression levels of murine and hDNASE1 by direct comparison of (E) cell extracts four hours and of (F) SN 24 hours after induction by DPZ. Whereas in extracts the difference is 2–3 times, extracellularly (SN) it is ~ 70 times. Marker: PageRuler™ Prestained Protein Ladder. Data shown for one exemplary experiment employing clone 1 for each preOst1-mDNase1 and -hDNASE1^co^ (Fig 6).

Comparing translation with transcription, we found that DNase1 protein was already detectable one hour after transfer of the cells into the expression medium as deduced by DPZ of extracts from induced and non-induced cells (Fig 8A and B).

The fact that mRNA and protein rose in parallel points to a short lag phase between transcription and translation. Correlating with transcription, translation occurred to a higher extend after MeOH induction (Fig 8A and B). It must be noted, that for the detection of DNase1 in non-induced cells, the gels had to be incubated at a higher temperature, which promoted nuclease activity mimicking an elevated protein amount. Interestingly, the turning point of translation occurred early after four hours, i.e., two hours later than transcription. With ongoing time, translation dropped continuously for both orthologues (Fig 8A and B).

When comparing the kinetics of intra- with those of the extracellular DNase1 occurrence, we found that secreted DNase1 was first detectable in the SN after two hours, i.e., one hour after the beginning of translation, as deduced by HCA for mDNase1 and DPZ for hDNASE1 (Fig 8C and D). Consistent to the kinetics of its intracellular occurrence, extracellular DNase1 activity rose over 24 hours. Although the kinetics of translation were similar for both, we found a general difference in the expression behavior and level. Thus, when comparing cell extracts at the timepoint of maximal translation after four hours, the intracellular amounts of both orthologues differed by only 2–3 times as estimated by DPZ (Fig 8E). In contrast, the maximal extracellular amounts found after 24 hours differed by ~ 70 times (Fig 8F). Since the detection sensitivity of DPZ for hDNASE1 is about threefold lower than for mDNase1 (see final experiment), we assume that the loading of the secretory compartment with hDNASE1 was similar to mDNase1, whereas the overall production differed by ~ 20 times. This may be due to a retarded transit of hDNASE1 through the rER.

Therefore, we tried to evaluate, whether the reduced production was accompanied by an UPR. As a marker, we analyzed the kinetic of functional *Hac1s* mRNA coding for a key transcription factor regulating UPR [65,96]. Apparently, in MeOH induced cells *Hac1s* occurred early after the first appearance of intracellular DNase1 (Fig 7E and F compared to Fig 8A and B). However, induction of *Hac1s* during hDNASE1 expression occurred to a higher extend and for a prolonged time, illustrating that UPR is a more limiting factor for hDNASE1 expression. Furthermore, in non-induced cells *Hac1s* was even induced during washing with PBS, which however occurred independently of the DNase1 orthologue expressed and therefore reflects a general stress response. After transfer of the cells into expression medium, *Hac1s* dropped and was reactivated only in hDNASE1 expressing cells after two hours, indicating, that even small amounts of hDNASE1 in contrast to mDNase1 induce an UPR (Fig 7E and F).

Finally, we evaluated long-term expression as a strategy to increase recombinant protein levels [59,97]. Therefore, the cells were cultivated for 72 hours at optimal conditions with a reduced cell density (OD_600_ =  40) to guarantee a prolonged nutrient supply and cell survival. Surprisingly, no enhanced expression occurred for both orthologues during 48 hours even after additional supply with MeOH (S6 Fig) suggesting that after an initial induction the cells enter a stationary phase with growth and DNase1 expression arrest. In contrast to hDNASE1, we found an increase of mDNase1 after 72 hours, which however was accompanied by the occurrence of cellular proteins suggesting initiation of cell death. This correlates with a reduced metabolism as deduced from a general downregulation of actin in MeOH treated as well as untreated cells (Fig 7C and D) and is in contrast with studies, which expressed recombinant proteins over longer time periods though with significantly lower cell densities [98].

### Purification of human and murine DNase1

After establishing expression, we purified DNase1 from culture SN. Thus, preOst1-mDNase1 and -hDNASE1^co^ were grown to OD_600_ =  6 in 0.5x BMGY at 28 °C, washed with PBS, and finally expressed at an OD_600_ =  40 in 1% (w/v) PM containing 2.5% (v/v) MeOH at 28 °C for 24 hours. Like described before [57], we first used DEAE chromatography as an initial step and eluted DNase1 at 150 and 200 mM NaCl (S7 Fig). In contrast, the protein of ~ 37 kDa, which overlayed the low amounts of hDNASE1 in SDS-PAGE, eluted at 250–500 mM NaCl as shown for SN of pPinkHC mock-transfected cells (S8 Fig). Since twice the volume of expression culture was used for hDNASE1, its DEAE elution pattern contained more background proteins compared to mDNase1. As described before, we found that DEAE chromatography followed by P-100 gel-filtration is insufficient to isolate pure DNase1 (S4 Fig). Therefore, the DEAE eluates with hDNASE1 were subjected to HAP chromatography with hDNASE1 eluting at ~ 50 mM sodium phosphate (S7 Fig). We omitted this step before gel-filtration for mDNase1, because the SN contained a purer protein of interest (S7 Fig). Subsequently, both nucleases were subjected to P-100 gel-filtration, isolated from the filtrates by HAP chromatography, dialyzed against, and kept in storage buffer pH 6 at 4 °C (S7 Fig). Protein concentrations were determined by the Bradford assay and counter calculated by comparing de-N-glycosylated samples on silver gels. Overall, ~ 0.4 mg pure mature rhDNASE1 compared to ~ 8 mg rmDNase1 per Liter expression culture (OD_600_ =  40) were purified.

### Biochemical comparison of recombinant DNase1

Finally, we evaluated similarities and differences of recombinant DNase1 expressed in *P. pastoris* with Pulmozyme®. Therefore, the pH optima, specific activities, inhibition by monomeric actin, the glycosylation pattern, as well as the refolding ability after denaturation were determined. Concerning the pH optimum, Pulmozyme® and rhDNASE1 behaved similar, i.e., showed a maximal activity at pH 7.0 and 7.5 in the presence of either Mg^2+^ or Mn^2+^ in addition to Ca^2+ ^, respectively (S9 Fig). The most obvious difference of Pulmozyme® and rhDNASE1 to rmDNase1 was for rmDNase1 a shift of the pH optimum by half a value to the basic direction (S9 Fig).

**Fig 9:**
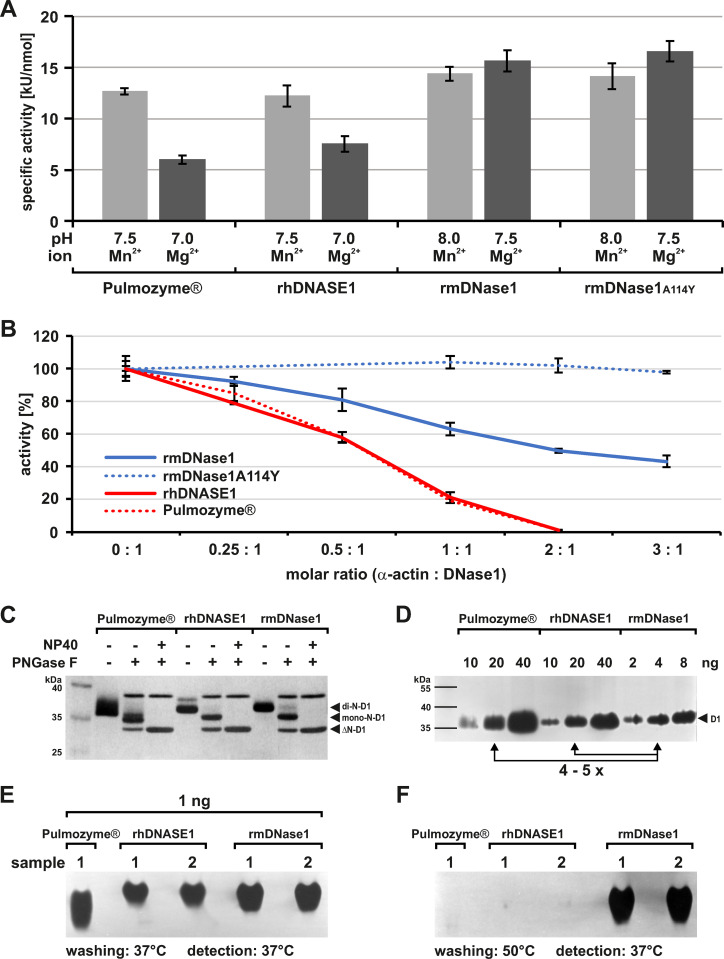
Comparison of DNase1 produced in *Pichia pastoris* with Pulmozyme®. (A) Determination of the specific activities at the pH optima (S9 Fig) in the presence of either Mg^2 +^ or Mn^2 +^ in addition to Ca^2 +^ by HCA. Pulmozyme® and rhDNASE1 with a clearly higher activity in the presence of Mn^2 +^ compared to Mg^2 +^ (each **p** <  0.001). In contrast, rmDNase1 shows less ion-specificity with a slightly raised activity in the presence of Mg^2 +^ (p =  0.0023). Overall, rmDNase1 is more active compared to rhDNASE1 most strikingly in the presence of Mg^2 +^ (for both cations **p** <  0.001). (B) Skeletal muscle α-actin inhibits rhDNASE1 completely at a molar ratio of 2:1 (α-actin: DNase1), whereas rmDNase1 is inhibited only to ~ 50% (p <  0.001) as evaluated by HCA. The rmDNase1^A114Y^ mutant lacks inhibition. The activity without actin was set 100% for each nuclease. (C) Analysis of N-glycosylation employing each 1 µg protein in the presence or absence of PNGase **F** (41 kDa) as evaluated by silver gel analysis. In contrast to Pulmozyme®, rhDNASE1 and rmDNase1 possess a di-N-glycosylation with a higher molecular mass (MM). After full de-N-glycosylation (ΔN-D1) the MM of human and rmDNase1 is equal (29.3 vs. 29.7 kDa). (D) Refolding ability in DPZ after DNase1 denaturation at 95 °C in the presence of β-mercaptoethanol. The refolding difference between rhDNASE1 and rmDNase1 is about 4–5 times as deduced from their specific activities in DPZ. (E) Compared to DPZ, the specific activity of rmDNase1 is only about two times higher than that of hDNASE1 in native PAGE zymography (NPZ). The NPZ gels were first, washed for pH adaption, and secondly, incubated for nuclease detection in buffer, pH 7.8–8 with Ca^2 + ^/Mn^2 +^ at 37 °C comparable to DPZ. (F) Washing the NPZ gels at 50 °C prior to detection led to a specific inactivation of hDNASE1 in NPZ. Marker: PageRuler™ Prestained Protein Ladder. Data shown for single exemplary experiments: Mean of each two DNase1 batches measured at least in duplicate.

In addition to the pH optima, the specific activities of rhDNASE1 and Pulmozyme® in the presence of Ca^2 + ^/Mn^2 +^ were identical (Fig 9A: 12.3 ±  1.0 vs. 12.7 ±  0.3 kU/nmol), whereas in the presence of Ca^2 + ^/Mg^2 +^ rhDNASE1 was ~ 25% more active (Fig 9A: 7.6 ±  0.8 vs. 6.0 ±  0.4 kU/nmol, p =  0.0017).

Again, rmDNase1 behaved differently. Compared to rhDNASE1 and Pulmozyme® its specific activity in the presence of Ca^2 + ^/Mn^2 +^ was ~ 13% (Fig 9A: 14.4 ±  0.7 kU/nmol, p <  0.001) and most strikingly with Ca^2 + ^/Mg^2 +^ it was even two times higher (Fig 9A: 15.7 ±  1.0 kU/nmol, p <  0.001) at the individual pH optima. Furthermore, and in contrast to rhDNASE1, which showed a significantly higher activity in the presence of Mn^2 +^ (each p <  0.001), rmDNASE1 showed a slight preference for Mg^2 + ^(p =  0.0023). No difference existed between WT and the actin resistant mutant rmDNase1^A114Y^ (Fig 9A).

Inhibition of the different DNase1 isoforms by monomeric skeletal muscle α-actin gave the expected results: First, Pulmozyme® and rhDNASE1 were fully inhibited at a molecular ratio of 2:1 (α-actin vs. hDNASE1), secondly, rmDNase1 could only be inhibited by ~ 50% (p <  0.001), and thirdly, the actin resistant mDNase1 mutant was not inhibited at all (Fig 9B).

The most notable difference between Pulmozyme® and DNase1 produced in *P. pastoris* is the glycosylation (Fig 9C). Although, all DNase1 isoforms were di-N-glycosylated as shown by sequential de-N-glycosylation with PNGase F, the molecular mass (MM) and type differed. Thus, Pulmozyme® possesses an N-glycosylation of lower MM, more inconsistent size and most likely of the complex type, which is typical for mammalian cells. In contrast, N-glycosylation by *P. pastoris* is of the high-mannose type [99] and more consistent, although a low amount of rhDNASE1 showed a glycosylation of higher MM (Fig 9C), whereas rmDNase1 was partially only mono-N-glycosylated and eluted exclusively in the HAP E50 fraction (S10 Fig). This phenomenon points to an expression maximum for mDNase1 in *P. pastoris* as already observed [57]. Consistent with published data [34], mono- has a lower specific activity than di-N-glycosylated rmDNase1, which preferentially eluted in HAP E25. Since purified rmDNase1 is a mixture of mono- with a dominance of the di-N-glycosylated form, the specific activity is lower than the pure di-N-glycosylated form (S10 Fig, p =  0.0147). After de-N-glycosylation, all proteins showed the expected MM of 29.7 (rmDNase1) and 29.3 kDa (rhDNASE1) (Fig 9C).

During our experiments, we noticed a different detection sensitivity of the DNase1 orthologues by DPZ. Thus, when the purified nucleases were analyzed at nearly optimal conditions for both (Ca^2 + ^/Mn^2 + ^, pH 7.8–8.0), the detection sensitivity for rmDNase1 was 4–5 times higher than for rhDNASE1, i.e., 4–5 times more rhDNASE1 had to be employed to reach similar signals (Fig 9D), whereas in native PAGE zymography (NPZ) the difference was only about two times (Fig 9E). Since in DPZ proteins are denatured by heat and β-mercaptoethanol, the remaining detection difference between DPZ and NPZ hints to an about three times lower re-folding ability towards a native structure of Pulmozyme® and rhDNASE1 compared to rmDNase1. Furthermore, we found a difference in the thermostability of both orthologues in NPZ. Thus, performing pH adaption of the NPZ gels at 50 instead of 37 °C prior to nuclease detection led to an inactivation of solely hDNASE1 (Fig 9F). Concludingly, rhDNASE1 possesses a lower renaturation ability in DPZ and a higher denaturation sensitivity in NPZ compared to rmDNase1 which clearly points to thermodynamic structural differences between both orthologues.

## Discussion

### Comparative expression of human and murine DNase1

The aims of the study were the expression of hDNASE1 in *P. pastoris* analogous to mDNase1 [57] and a biochemical comparison of both identically produced orthologues. Expression by yeast appears simple and we wondered why this had not yet been done for hDNASE1*.* Unexpectedly, its expression using the αMF-SP resulted in a 70–80 times lower yield compared to mDNase1, which cannot be explained by extracellular degradation or by cytotoxicity. Cytotoxicity appears unlikely in view of the equal expression of WT and actin-resistant mDNase1 and a reduced of the inactive isoform. Our data implicate that no functional DNase1 is in the cytoplasm, probably due to the binding of chaperons assisting post-translational import. To our knowledge, this is the first study describing such a drastic expression difference of two orthologues, which possess an amino-acid sequence with 81.8% homology and 89.8% similarity and comparable structural/functional features. Nevertheless, despite the homology they apparently expose different folding abilities to reach native state, which is supported by the findings that hDNASE1 has a much lower specific activity compared to mDNase1 in DPZ than in NPZ, that it is more sensitive to thermal inactivation in NPZ, and that it particularly induces an overactivated UPR. Protein folding appears to limit DNase1 expression in *P. pastoris* in general, since even a loss-of-function point mutation acts disturbing. Our data support the importance of the native structure for the fate of a protein, i.e., for its secretion or degradation [100]. Unequal folding can result in intermediates, which form at varying temperatures and influence differentially the progression through the secretory compartment [101,102]. An individual temperature effect of a change from post- to co-translational import was indeed detected, supporting a different folding and probably aggregation behavior. For hDNASE1 this overall led to a significantly lower production and it was difficult to detect it in SN, a problem aggravated by an impaired maturation. Previously we solved this by incubating dialyzed SN containing αMF-mDNase1 at 37°C which led to pro-peptide procession by protease(s) and/or glycosylase(s) released from yeast [57]. Obviously, this did not impair mDNase1 as proven by identical specific activities of rmDNase1 produced in both studies. Inefficient maturation might be caused by hyper-tri-N-glycosylation of the pro-peptide hindering Kex2 to cleave the C-terminal dibasic Arg-Lys spacer-peptide at the transition to DNase1. Similar results exist for an insulin precursor [103], onconase [104], as well as for mDNase1L2 and mDNase1L3 [57].

To improve maturation, we deleted the αMF pro-peptide [104]. Fortunately, use of only the pre-peptide led to secretion of pure mature DNase1. Unfortunately but consistent with results obtained by this deletion, mDNase1 production decreased threefold [105,106] which is most likely explained by two pro-peptide functions, i.e., support of post-translational rER import and of Erv29p receptor mediated protein transfer to the GA [73,77,107]. In contrast, hDNASE1 expression remained low due to an ineffective post-translational import, which cannot be improved by the pro-peptide. Since after a switch to co-translational import mDNase1 production was still higher, further factors preferentially limiting hDNASE1 expression must exist. Indeed, hDNASE1 induces a more pronounced unproductive UPR. Thus, three bottlenecks exist for DNase1 expression in *P. pastoris*. Two of them, import and post-translational processing in the rER, differ in their consequence between both orthologues probably due to individual folding abilities. The Erv29p dependent receptor mediated transfer is however only indirectly dependent on folding since it solely acts on native proteins with an N-terminal pro-peptide. The more protein is native, the less UPR induced degradation occurs, and the more protein is transferred and finally secreted. Due to the conserved structure of both DNase1 molecules one can assume that after reaching native state they are at least similarly transferred. Lack of this might however lead to a production limiting disturbance, which cannot be prevented by a switch to cotranslational rER import.

Consistent with published results, increasing nutrients and cultivation temperature promoted expression independently of the SP used [93,108,109], although temperature can also act oppositely [110,111]. The effect probably results from a generally enhanced metabolism supporting the secretory pathway. A raise of MeOH had an additional SP independent general positive effect but also specifically compensated ~ 13% of the decrease caused by the pro-peptide deletion. This implies that an increased energy supply partially induces substitutive co-factors for the different steps of the secretory pathway. The general improvement by MeOH agrees well with published results [59,112–114]. By changing post- to co-translational import additional ~ 22% and thus a total of approximately half of the threefold decrease in mDNase1 expression caused by the pro-peptide deletion was compensated when comparing the results using the entire αMF- with those of the Ost1-SP under optimized conditions. However, a third of the decline remained apparently due to a less efficient constitutive compared to a receptor-mediated transfer.

In summary, a maximal expression level exists for DNase1 in *P. pastoris* most likely defined by the folding behavior and co-factors supporting folding and transfer. Our data show that expression is downregulated at the transcriptional level and/or by a translational arrest, when the capacity of the single steps of the secretory pathway are reached in the absence of sufficient energy [115,116]. For mDNase1 this capacity is higher than for hDNASE1, but for both it is reached with a single transgene expression cassette and cannot be elevated by a gene dose effect. Although some parameters must have increased hDNASE1 expression to a higher extend, the combination of all did however not lead to the yield obtained for mDNase1. At the end of our optimization efforts, an expression difference of ~ 20 times remained, though an improvement by four times was achieved resulting in ~ 8 mg pure mature rmDNase1 vs. ~ 0.4 mg rhDNASE1 per Liter expression medium of a culture with a cell density of OD_600_ =  40 in 24 hours. This appears marginal compared to hyperactive PASylated rhDNASE1 expressed in a modified CHO-K1 tumor cell line [56]. Minus the MM of the PAS-fusion part, ~ 374 mg/L rhDNASE1 are produced in 14 days comparable to WT rhDNASE1 [55].

Although these studies might question our attempts to produce rhDNASE1 in *P. pastoris*, they cannot be used as a counter-argument due to several reasons. Most important are the production time and the cultivation regime. Our study is a lab bench project to evaluate the production possibility as well as functionality of rhDNASE1. To achieve commercially usable quantities, further methods are available for yeast. The overactivated UPR might, e.g., be solved by co-expression of suitable chaperones or by adapting the UPR intensity [66], whereas the combined growth and expression arrest could be modified in a fermenter setup allowing a prolonged expression with refeeding and induction [117]. Here second-generation *P. pastoris* strains could be employed using renewable carbon sources or industrial waste products like glycerol and lignocellulose [118]. Even the replacement of toxic MeOH by using an ethanol inducible AOX1 or other promoters is plausible [119]. Furthermore, N-glycosylation could be humanized by Glycoswitch^®^ [120]. In conclusion, progress can be made to reach comparable production yields to CHO cells. Therefore, an optimal equilibrium of transcription and energy supply for post-transcriptional processes and cell metabolism must be reached. The advantages of yeast are still worthwhile. Low production costs, high cell densities in fermenter scales, fast growth, and importantly a production without potential human pathogenic viruses in the absence of mammalian oncogenes still make yeast to the most used organism for recombinant protein expression [59,119].

### Biochemical features of murine and human DNase1

The suitability of yeast for the production of rhDNASE1 is proven by our biochemical analysis. Thus, rhDNASE1 shows the same activity profile with respect to pH and cation dependence as well as an identical actin inhibition as Pulmozyme® or rhDNASE1 expressed in HeLa S3 cells [54]. Furthermore, the specific activity of rhDNASE1 is almost equal to that of Pulmozyme® at optimal pH for each co-cation, i.e., Mg^2 +^ or Mn^2 +^ in addition to Ca^2 + ^. The specific activity of rhDNASE1 is higher when using Mn^2 +^ which can be explained by a shift from single- to double-stranded cutting resulting in a faster DNA digestion [121,122]. The underlying molecular mechanism is not resolved yet [46] and the fact that mDNase1 shows almost equal activities in the presence of Mg^2 +^ or Mn^2 +^ makes the theory questionable. The main difference between rhDNASE1 produced in yeast and Pulmozyme® is the glycosylation. While Pulmozyme® has sugar trees with a greater variety of monomers, N-glycosylation of rhDNASE1 expressed in *P. pastoris* is more uniform with a slightly higher MM. This results from a consistent mannose-rich N-glycosylation performed by yeast compared to the complex type in mammalian cells [59]. A small portion of rhDNASE1 shows an N-glycosylation of higher MM, which might result from its prolonged dwell time accompanied by an ongoing extension of the sugar trees in the rER. After de-glycosylation however, all proteins showed the expected MM proving the correct cleavage of the Ost1-SP without N-terminal extensions.

By comparing murine with rhDNASE1 produced under identical conditions we show for the first time, that rmDNase1 is twice as active in the presence of Mg^2 + ^, a phenomenon which does not exist to this extend when using Mn^2 + ^. Seven point-mutations with insertion of basic amino acids into or near to the catalytic center of hDNASE1 have been performed to create a hyperactive isoform in the presence of Mg^2 +^ [123]. Two of these, Q9R and N74K, naturally occur in mDNase1 and might explain the difference in their specific activity. Of note, only a marginal difference exists for the specific activity of rmDNase1 when using Mg^2 +^ or Mn^2 + ^. Thus, we postulate that the activity difference of rhDNASE1 using the individual cations might be caused by the lack of R9 and K74. It will be interesting to investigate whether like for rmDNase1 the specific activity of hyperactive rhDNASE1 is equal in the presence of Mg^2 +^ and Mn^2 + ^. Concerning the pH optimum, we found a shift by half a magnitude to the basic direction for rmDNase1. Whether this reflects an adaption to differences in the pH of body fluids between man and mice remains speculative. A further difference is their varying inhibition by monomeric actin. Although both enzymes possess identical binding sites for actin, the maximal inhibition of rmDNase1 by G-actin is only 50% of that of rhDNASE1 [57]. From the differences described for both orthologues it can be concluded that despite their high homology they vary to a certain extend in the architecture and function of their catalytic center.

Evaluating differences between orthologues might help to optimize therapeutic biologics with respect to biochemical or physiological requirements. In principle, a targeted transfer of some abilities from murine to hDNASE1 would enhance its effectiveness. Indeed, actin-resistant hyperactive rhDNASE1 avoiding depolymerization of F-actin and inhibition of administered rhDNASE1 by liberated G-actin appears to be an optimal therapeutic improvement not only in CF [49,123,124] but also under conditions of excessive NET formation as induced for instance by SARS-CoV-2 virus.

## Supporting information

S1 Fig Signal peptides used for DNase1 expression. (A) Amino acid sequence of αMF-SP in vector pPinkα-HC with the following differences to the αMF-SP of *S. cerevisiae* (UniProtKB Acc. No. P01149): L40S, D83E, and lack of the C-terminal spacer EAEA tetrapeptide. (B) Amino acid sequence of the Ost1-SP of *S. cerevisiae* (UniProtKB Acc. No. P41543) used for expression in combination with vector pPinkHC (S1 File).
(TIF)

S2 Fig Influence of nutrients and cultivation temperature on preαMF-hDNASE1 expression. Semi-quantitative analysis of SN with DPZ. (A) Variation of the growth medium combined with the so far used expression medium of 0.5% (w/v) PM with 0.5% (v/v) MeOH shows a rising induction with increasing nutrients in the growth medium at 24 and 28 °C. (B) Variation of the peptone amount in the expression medium combined with the optimal 0.5x to 1x BMGY growth medium led to a saturated expression at 1% PM using 0.5% MeOH at 28 °C. (C) By comparing all optimized parameters (growth- and expression medium as well as cultivation temperature) only marginal differences are detectable with a tendency to a higher expression at 28 °C. Marker: Prestained protein marker PAN-Biotech™. Data shown for single exemplary experiments using preαMF-hDNASE1 clone 2 (Fig 3D).
(TIF)

S3 Fig Influence of nucleolytic cytotoxicity on preαMF-DNase1 expression. Inactivation of DNase1 by a D168S mutation with no positive effect on the expression level of (A) preαMF-mDNase1 and (B) -hDNASE1co as evaluated by Coomassie gel analysis of SN. The loss-of-function mutation in the catalytic center is proven by DPZ of SN below. Marker: Prestained protein marker PAN-Biotech™. Data shown for single exemplary experiments with one to three clones as indicated (numbers of the preαMF-DNase1 WT clones refer to Fig 3).
(TIF)

S4 Fig Factors influencing intra- and extracellular DNase1 protein stability. (A, B) Adapting the pH of the PM medium reveals optimal expression and stability of preαMF-mDNase1 and -hDNASE1co at pH 6-7 as shown by (A) HCA (pH 5 vs. 6, p <  0.001 and pH 7 vs. 8, p <  0.001) and (B) semi-quantitative silver gel analysis of SN combined with DPZ below. (C, D) Addition of the serine protease inhibitor AEBSF or protease inhibitor cocktail (PIC) had no positive effect, implicating lack of extracellular proteolytic DNase1 degradation as evaluated by (C) HCA and (D) silver gel analysis combined with DPZ below. (E, F) Comparison of preαMF-DNase1 expression by different strains of *PichiaPink™ pastoris*. Strain 1: WT, strain 2: lack of proteinase A and carboxypeptidase Y, strain 3: lack of proteinase B, and strain 4: lack of all three proteases [78]. In contrast to preαMF-hDNASE1^co^ expression with no specificity, preαMF-mDNase1 was optimally and comparably expressed with strain 1 and 4 as shown for (E) mDNase1 by HCA for each two clones of strain 1 - 3 and exemplary clone 2 of strain 4 (Fig 3A) and for (F) hDNASE1 by silver gel analysis combined with DPZ below for each two clones of strain 1 - 3 and exemplary clone 3 of strain 4 (Fig 3F). (G) Comparative HCA of original and purified Pulmozyme® added to a culture of *PichiaPink™ pastoris* strain 4 mock-transfected with vector pPinkHC and incubated for 24 hours at 28 °C. The specific activity of purified Pulmozyme® estimated by the total protein amount of the isolate as determined by the Bradford assay implicates a strong impairment (~32% difference to original Pulmozyme®, p <  0.001). Correction of the protein amount by an alignment based on the activity of original Pulmozyme® however, points to an impure isolate due to an insufficient purification procedure with only a marginal impairment of Pulmozyme® by the culture conditions (~9% difference, p <  0.001). The alignment was checked and counter calculated based on (H) densitometrical silver gel analysis of each 1 µg de-N-glycosylated protein. Marker: Prestained protein marker PAN-Biotech™ in (B, F) and PageRuler™ Prestained Protein Ladder in (D, H). Data shown for single exemplary experiments.
(TIF)

S5 Fig Gene dose effect on preOst1-DNase1 expression. (A) Pilot expression of preOst1-mDNase1 employing two (2x) compared to one (1x) expression cassette with a slightly positive gene dose effect at 24 °C when using suboptimal 0.5% (v/v) MeOH as evaluated by silver gel analysis of SN. (B) Mean mDNase1 activity in the SN of the clones shown in (A) as determined by HCA reveals the low increase at non-induced (p =  0.0158) and induced conditions (p <  0.001). (C) The positive effect disappeared at optimized conditions, i.e., by raising the cultivation temperature to 28 °C and increasing MeOH to 2.5% as determined by HCA of SN derived from each clone 1 (p =  0.0068). (D) Comparable pilot- as well as (E) optimized expression of hDNASE1^co^ with a similar result as evaluated by DPZ of SN derived from each clone 1. Marker: PageRuler™ Prestained Protein Ladder. Data shown for single exemplary experiments with one to three clones as indicated (numbers of the clones with a single expression cassette refer to Fig 6).
(TIF)

S6 Fig Evaluation of long-term preOst1-DNase1 expression. Prolonged expression of (A) murine and (B) human preOst1-DNase1 over 72 hours with (+) or without (-) daily application of 2.5% (v/v) MeOH with no ongoing DNase1 expression as evaluated by silver gel analysis of SN combined with (A) HCA or (B) DPZ below. Cells were incubated in 1% (w/v) PM at 28 °C and lowered cell density (OD_600_ =  40) to guarantee a prolonged nutrient supply and cell survival. After 72 hours an increased nucleolytic activity in the SN of preOst1-mDNase1 expressing cells could be detected (p <  0.001), however, accompanied by the occurrence of cellular proteins suggesting beginning cell death. Marker: PageRuler™ Prestained Protein Ladder. Data shown for single exemplary experiments using clone 1 for each preOst1-mDNase1 and -hDNASE1^co^ (Fig 6).
(TIF)

S7 Fig Purification of DNase1. Purification of recombinant mDNase1 (left column) and hDNASE1 (right column) from SN of preOst1-DNase1 expressing *P. pastoris*. (A, B) DEAE chromatography of dialyzed SN (DL =  dialysate, FT =  flow-through) with elution of DNase1 at 150 (E150) and 200 mM NaCl (E200) as evaluated for (A) mDNase1 by silver gel analysis and HCA and (B) hDNASE1 by silver gel analysis and DPZ below. (C) HAP chromatography (WB =  washing buffer, FT =  flow-through) of the DEAE eluates containing hDNASE1 with its elution at 50 mM sodium phosphate, pH 6.8 (E50) as shown by silver gel analysis. (D, E) Silver gel analysis of P-100 gel-filtration of the DEAE eluates E150 and E200 (mDNase1) or of the HAP eluate E50 (hDNASE1) shown in (A) and (C), respectively. (F, G) Silver gel analysis of the final HAP chromatography using the DNase1 containing gel-filtration fractions shown in (D) and (E), respectively. Marker: PageRuler™ Prestained Protein Ladder. Data shown for single exemplary experiments using clone 1 for each preOst1-mDNase1 and -hDNASE1^co^ (Fig 6).
(TIF)

S8 Fig Expression culture supernatant of control cells. Employing SN from vector pPinkHC mock-transfected *PichiaPink™ pastoris* strain 4 in DEAE chromatography shows that a protein of ~ 37 kDa co-migrating with DNase1 eluted at 250-500 mM NaCl. However, it did not interfere with the purification of DNase1, which eluted at 150 and 200 mM NaCl (S7 Fig) as evaluated by silver gel analysis (FT =  flow-through). Marker: PageRuler™ Prestained Protein Ladder. Data shown for one exemplary experiment.
(TIF)

S9 Fig Characterization of the DNase1 pH optima. Analysis of the relative DNase1 activity of (A) Pulomzyme®, (B) rhDNASE1, and (C) rmDNase1 with the HCA at different pH in the presence of 0.1 mM CaCl_2_ combined with either 1 mM MgCl_2_ or MnCl_2_. The highest activity measured for each nuclease was set 100%. Pulmozyme® and rhDNASE1 produced in *P. pastoris* behave similarly. Compared to Pulmozyme® and rhDNASE1, the pH optimum of rmDNase1 is shifted by half a value to the basic direction independently of the co-ion used in addition to Ca^2 + ^. Data shown for one exemplary experiment: mean of each two DNase1 batches measured at least in duplicate.
(TIF)

S10 Fig Sequential elution of mono- and di-N-glycosylated rmDNase1 from hydroxyapatite. (A) Elution profile of HAP chromatography with rmDNase1 after P-100 gel-filtration as evaluated by silver gel analysis. In contrast to the experiment presented in S7 Fig, an elution step with 25 mM sodium phosphate, pH 6.8 (E25) was included. The eluate E25 contained only di-N-glycosylated rmDNase1 whereas E50 was a mixture of mono- and di-N-glycosylated rmDNase1. (B) Specific activity of rmDNase1 in E25 and E50 as determined by HCA pointing to a reduced activity of the mono-N-glycosylated isoform as evaluated by the difference between the pure di-N-glycosylated E25 vs. the mixed E50 eluate (p =  0.0147). (C) Evaluation of the N-glycosylation pattern and amount of rmDNase1 present in E25 and E50 compared to Pulmozyme®. Each 1 µg protein was treated with PNGase F compared to its untreated control and analyzed by a silver gel. Marker: PageRuler™ Prestained Protein Ladder. Data shown for one exemplary experiment.
(TIF)

S1 File Sequence of vector pPinkHC. SnapGene file of the vector pPinkHC used in this work with an adapted multiple cloning site compared to the origin of Thermo Fisher - Invitrogen.
(DNA)

S2 File Vector maps of pPinkHC/preαMF-mDNase1. Maps of vector pPinkHC/preαMF-mDNase1 with a (A) single and (B) double (2x) expression cassette used in this study. Comparable vectors were employed for hDNASE1. Essential features are labeled. The transcription direction of both cassettes is identical. Vectors were linearized by *Afl*II cleavage prior to transformation.
(TIF)

S3 File Comparison of wildtype and codon optimized hDNASE1 (hDNASE1^co^). The nucleotide sequence for mature hDNASE1 without its natural N-terminal signal sequence of 22 amino acids was taken from GenBank (Acc. No. NM_005223) and codons occurring with a frequency of lower than 25% in *P. pastoris* were manually revised to the most used ones to generate a codon optimized (co) *hDNASE1*^*co*^ cDNA [91].
(PDF)
