# Supplementary figures and images for: Different results despite high homology: Comparative expression of human and murine DNase1 in Pichia pastoris
Source: PLoS One. 2025 Apr 29;20(4):e0321094. doi: 10.1371/journal.pone.0321094 (PMC12040185; doi:10.1371/journal.pone.0321094)

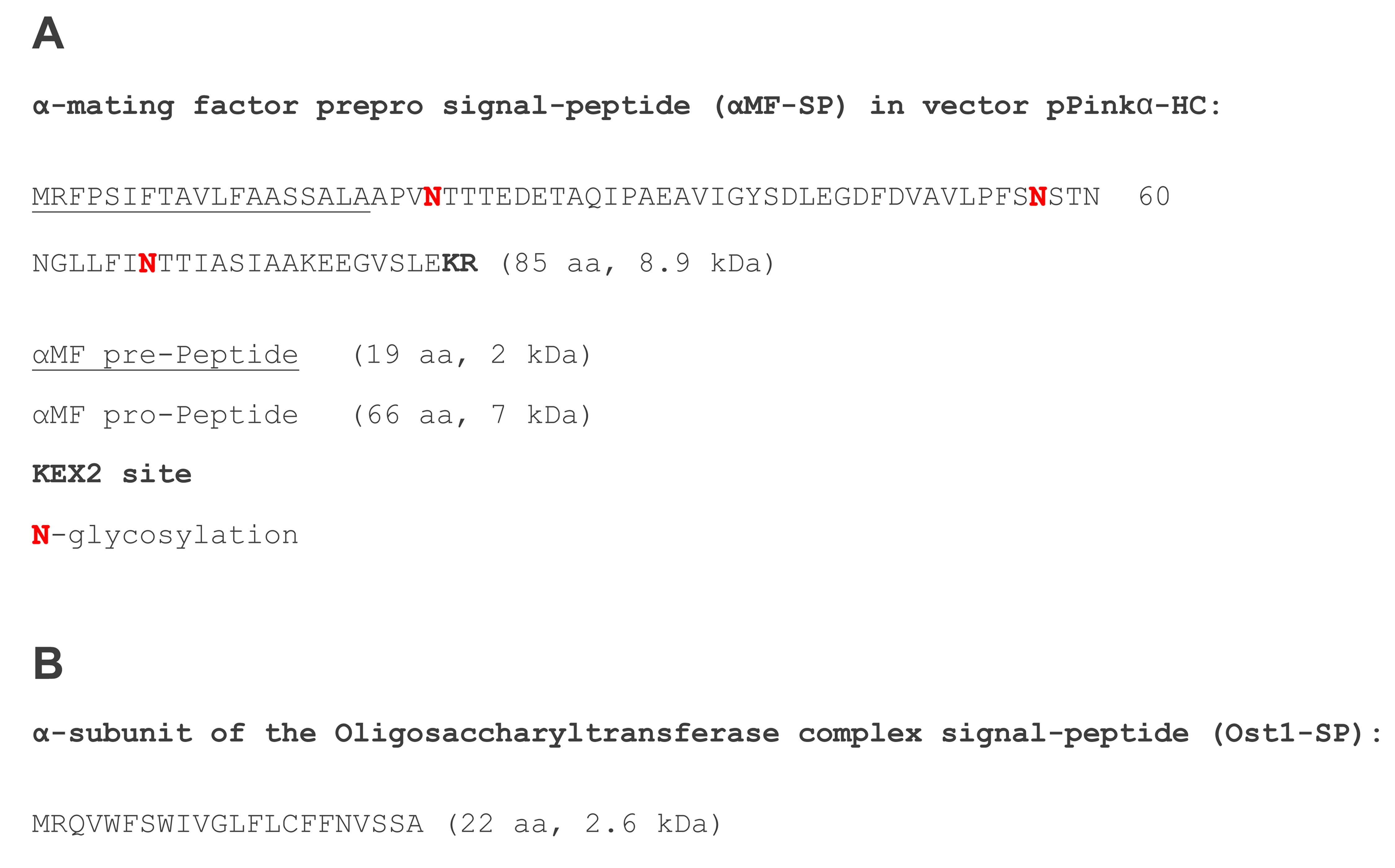

Supplement: S1 Fig — (A) Amino acid sequence of αMF-SP in vector pPinkα-HC with the following differences to the αMF-SP of S. cerevisiae (UniProtKB Acc. No. P01149): L40S, D83E, and lack of the C-terminal spacer EAEA tetrapeptide. (B) Amino acid sequence of the Ost1-SP of S. cerevisiae (UniProtKB Acc. No. P41543) used for expression in combination with vector pPinkHC (S1 File). (TIF) [file pone.0321094.s001.tif]

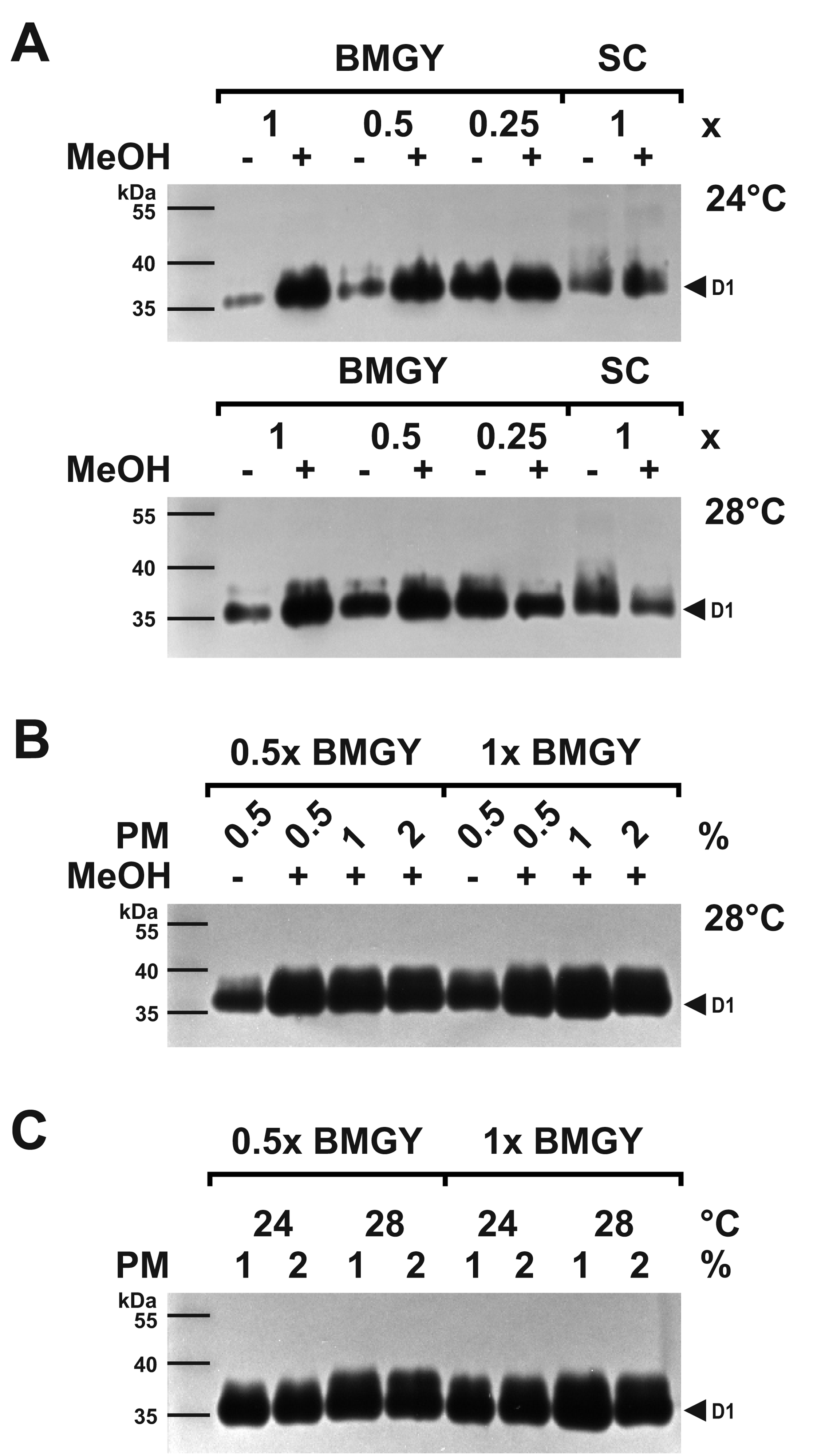

Supplement: S2 Fig — Semi-quantitative analysis of SN with DPZ. (A) Variation of the growth medium combined with the so far used expression medium of 0.5% (w/v) PM with 0.5% (v/v) MeOH shows a rising induction with increasing nutrients in the growth medium at 24 and 28 °C. (B) Variation of the peptone amount in the expression medium combined with the optimal 0.5x to 1x BMGY growth medium led to a saturated expression at 1% PM using 0.5% MeOH at 28 °C. (C) By comparing all optimized parameters (growth- and expression medium as well as cultivation temperature) only marginal differences are detectable with a tendency to a higher expression at 28 °C. Marker: Prestained protein marker PAN-Biotech™. Data shown for single exemplary experiments using preαMF-hDNASE1 clone 2 (Fig 3D). (TIF) [file pone.0321094.s002.tif]

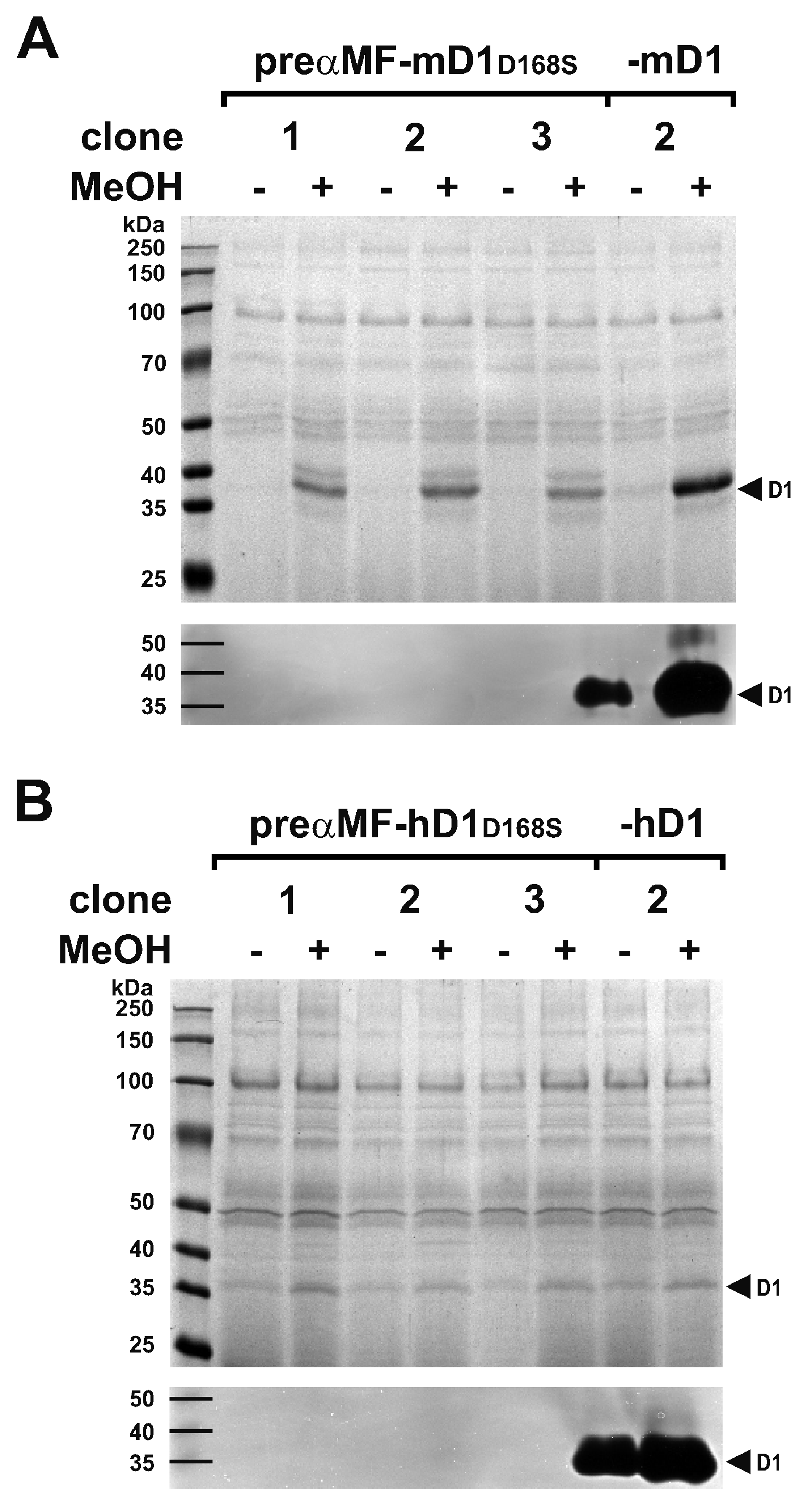

Supplement: S3 Fig — Inactivation of DNase1 by a D168S mutation with no positive effect on the expression level of (A) preαMF-mDNase1 and (B) -hDNASE1co as evaluated by Coomassie gel analysis of SN. The loss-of-function mutation in the catalytic center is proven by DPZ of SN below. Marker: Prestained protein marker PAN-Biotech™. Data shown for single exemplary experiments with one to three clones as indicated (numbers of the preαMF-DNase1 WT clones refer to Fig 3). (TIF) [file pone.0321094.s003.tif]

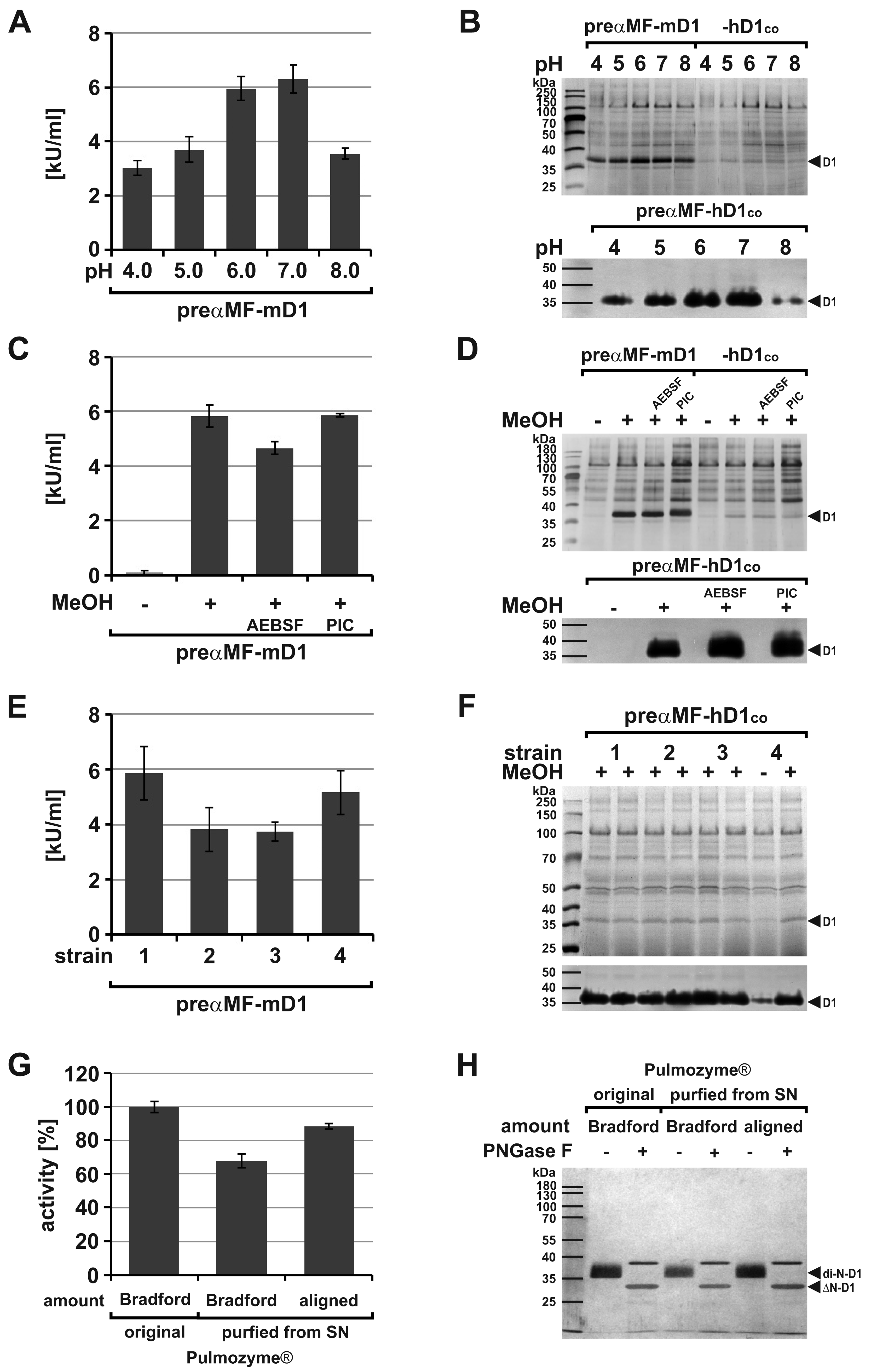

Supplement: S4 Fig — (A, B) Adapting the pH of the PM medium reveals optimal expression and stability of preαMF-mDNase1 and -hDNASE1co at pH 6-7 as shown by (A) HCA (pH 5 vs. 6, p < 0.001 and pH 7 vs. 8, p < 0.001) and (B) semi-quantitative silver gel analysis of SN combined with DPZ below. (C, D) Addition of the serine protease inhibitor AEBSF or protease inhibitor cocktail (PIC) had no positive effect, implicating lack of extracellular proteolytic DNase1 degradation as evaluated by (C) HCA and (D) silver gel analysis combined with DPZ below. (E, F) Comparison of preαMF-DNase1 expression by different strains of PichiaPink™ pastoris. Strain 1: WT, strain 2: lack of proteinase A and carboxypeptidase Y, strain 3: lack of proteinase B, and strain 4: lack of all three proteases [78]. In contrast to preαMF-hDNASE1co expression with no specificity, preαMF-mDNase1 was optimally and comparably expressed with strain 1 and 4 as shown for (E) mDNase1 by HCA for each two clones of strain 1 - 3 and exemplary clone 2 of strain 4 (Fig 3A) and for (F) hDNASE1 by silver gel analysis combined with DPZ below for each two clones of strain 1 - 3 and exemplary clone 3 of strain 4 (Fig 3F). (G) Comparative HCA of original and purified Pulmozyme® added to a culture of PichiaPink™ pastoris strain 4 mock-transfected with vector pPinkHC and incubated for 24 hours at 28 °C. The specific activity of purified Pulmozyme® estimated by the total protein amount of the isolate as determined by the Bradford assay implicates a strong impairment (~32% difference to original Pulmozyme®, p < 0.001). Correction of the protein amount by an alignment based on the activity of original Pulmozyme® however, points to an impure isolate due to an insufficient purification procedure with only a marginal impairment of Pulmozyme® by the culture conditions (~9% difference, p < 0.001). The alignment was checked and counter calculated based on (H) densitometrical silver gel analysis of each 1 µg de-N-glycosylated protein. Marker: Prestained [file pone.0321094.s004.tif]

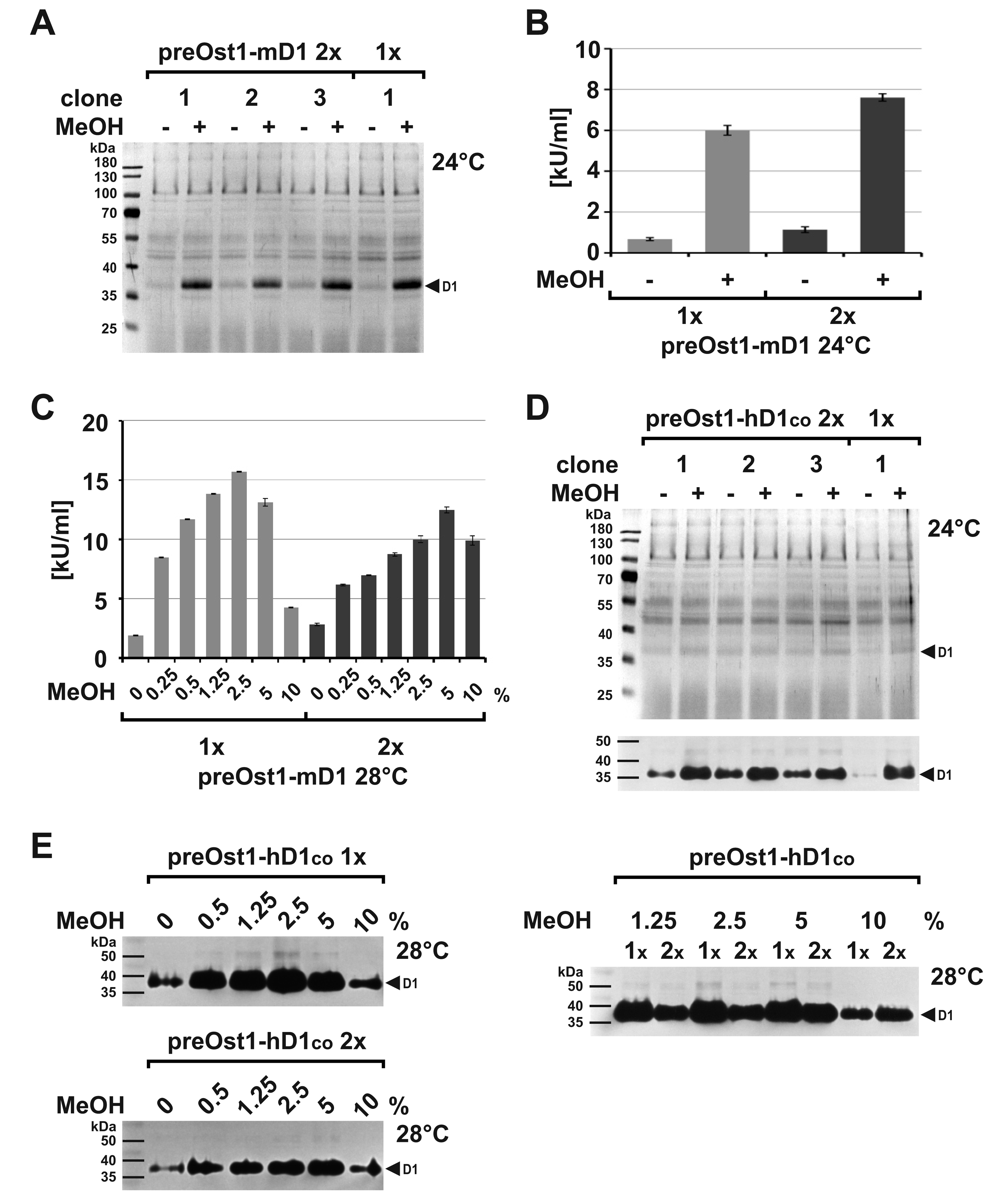

Supplement: S5 Fig — (A) Pilot expression of preOst1-mDNase1 employing two (2x) compared to one (1x) expression cassette with a slightly positive gene dose effect at 24 °C when using suboptimal 0.5% (v/v) MeOH as evaluated by silver gel analysis of SN. (B) Mean mDNase1 activity in the SN of the clones shown in (A) as determined by HCA reveals the low increase at non-induced (p = 0.0158) and induced conditions (p < 0.001). (C) The positive effect disappeared at optimized conditions, i.e., by raising the cultivation temperature to 28 °C and increasing MeOH to 2.5% as determined by HCA of SN derived from each clone 1 (p = 0.0068). (D) Comparable pilot- as well as (E) optimized expression of hDNASE1co with a similar result as evaluated by DPZ of SN derived from each clone 1. Marker: PageRuler™ Prestained Protein Ladder. Data shown for single exemplary experiments with one to three clones as indicated (numbers of the clones with a single expression cassette refer to Fig 6). (TIF) [file pone.0321094.s005.tif]

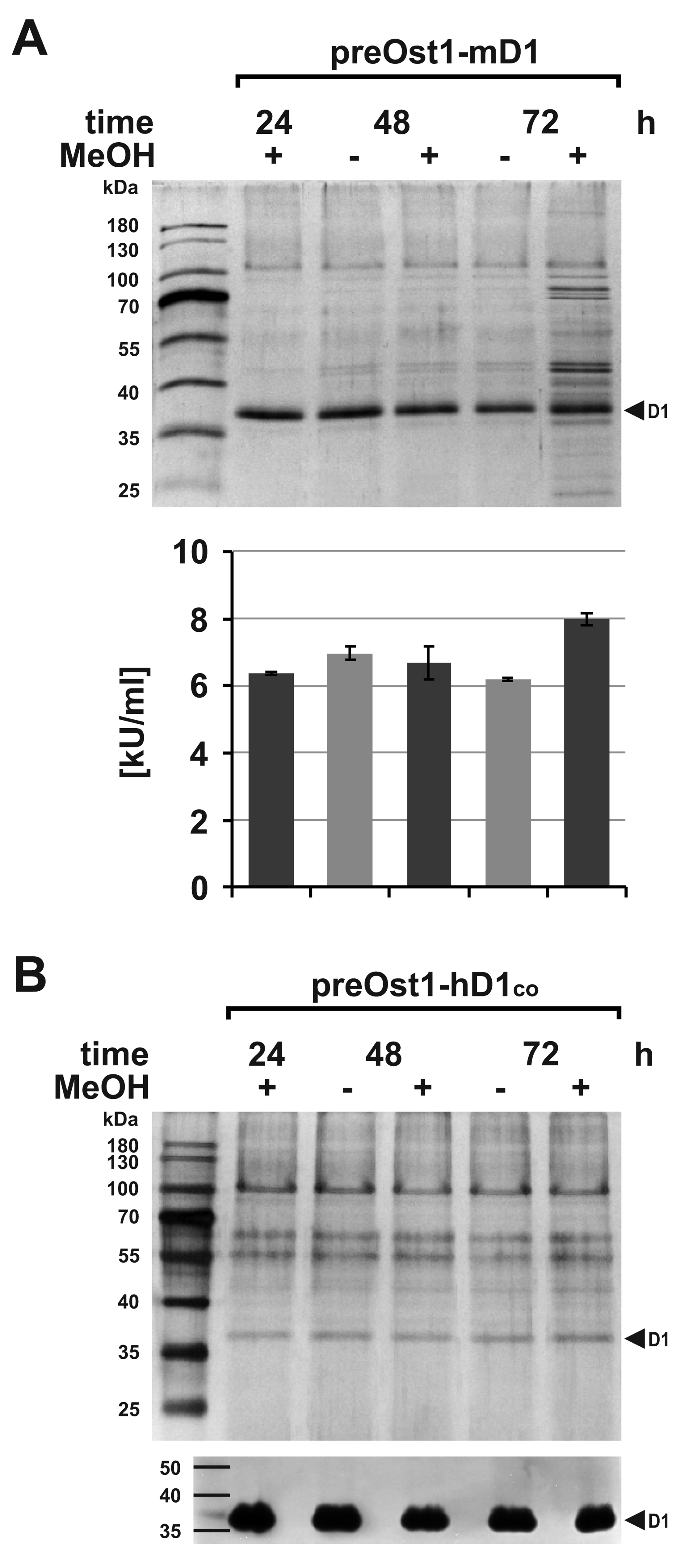

Supplement: S6 Fig — Prolonged expression of (A) murine and (B) human preOst1-DNase1 over 72 hours with (+) or without (-) daily application of 2.5% (v/v) MeOH with no ongoing DNase1 expression as evaluated by silver gel analysis of SN combined with (A) HCA or (B) DPZ below. Cells were incubated in 1% (w/v) PM at 28 °C and lowered cell density (OD600 = 40) to guarantee a prolonged nutrient supply and cell survival. After 72 hours an increased nucleolytic activity in the SN of preOst1-mDNase1 expressing cells could be detected (p < 0.001), however, accompanied by the occurrence of cellular proteins suggesting beginning cell death. Marker: PageRuler™ Prestained Protein Ladder. Data shown for single exemplary experiments using clone 1 for each preOst1-mDNase1 and -hDNASE1co (Fig 6). (TIF) [file pone.0321094.s006.tif]

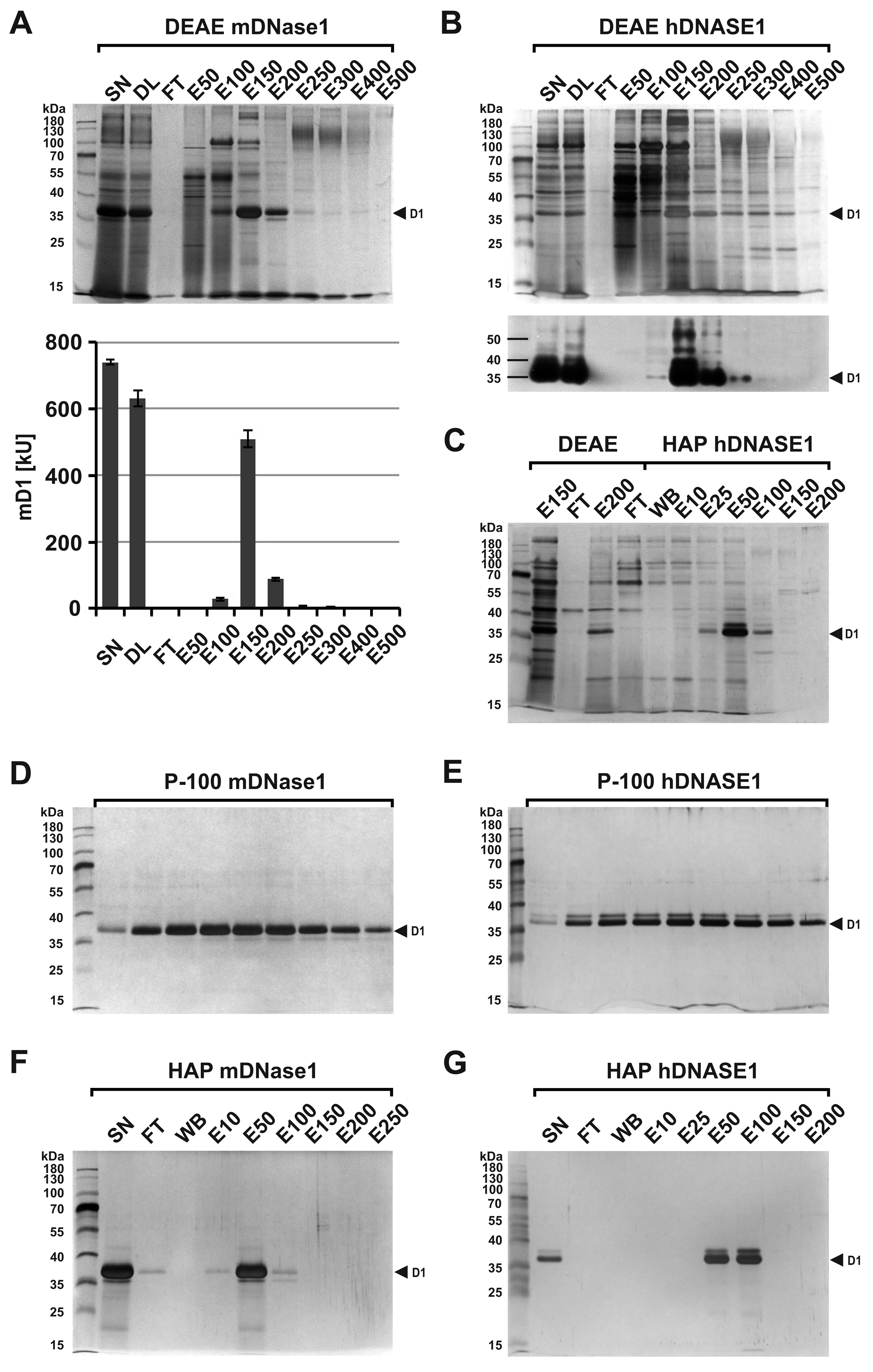

Supplement: S7 Fig — Purification of recombinant mDNase1 (left column) and hDNASE1 (right column) from SN of preOst1-DNase1 expressing P. pastoris. (A, B) DEAE chromatography of dialyzed SN (DL = dialysate, FT = flow-through) with elution of DNase1 at 150 (E150) and 200 mM NaCl (E200) as evaluated for (A) mDNase1 by silver gel analysis and HCA and (B) hDNASE1 by silver gel analysis and DPZ below. (C) HAP chromatography (WB = washing buffer, FT = flow-through) of the DEAE eluates containing hDNASE1 with its elution at 50 mM sodium phosphate, pH 6.8 (E50) as shown by silver gel analysis. (D, E) Silver gel analysis of P-100 gel-filtration of the DEAE eluates E150 and E200 (mDNase1) or of the HAP eluate E50 (hDNASE1) shown in (A) and (C), respectively. (F, G) Silver gel analysis of the final HAP chromatography using the DNase1 containing gel-filtration fractions shown in (D) and (E), respectively. Marker: PageRuler™ Prestained Protein Ladder. Data shown for single exemplary experiments using clone 1 for each preOst1-mDNase1 and -hDNASE1co (Fig 6). (TIF) [file pone.0321094.s007.tif]

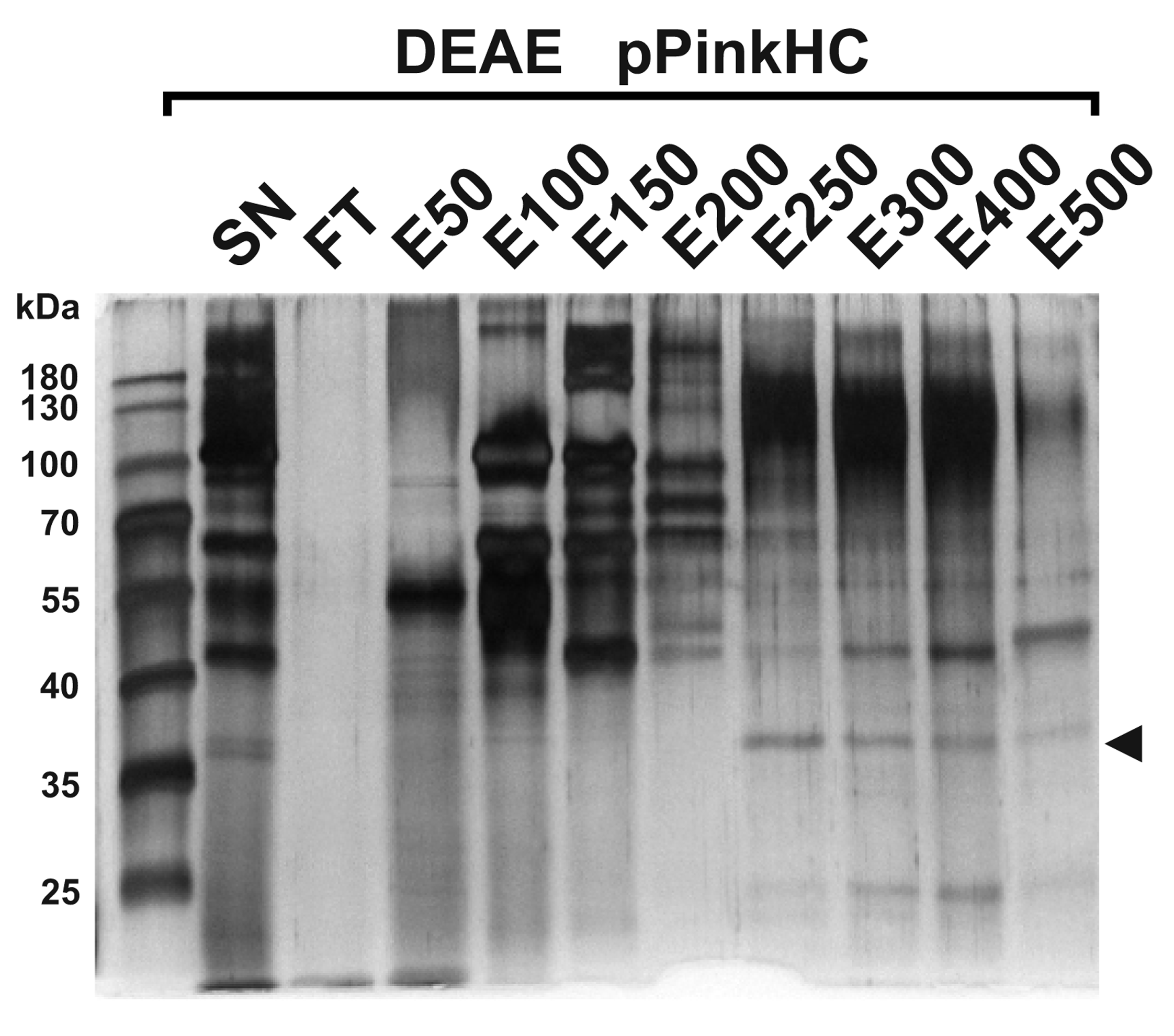

Supplement: S8 Fig — Employing SN from vector pPinkHC mock-transfected PichiaPink™ pastoris strain 4 in DEAE chromatography shows that a protein of ~ 37 kDa co-migrating with DNase1 eluted at 250-500 mM NaCl. However, it did not interfere with the purification of DNase1, which eluted at 150 and 200 mM NaCl (S7 Fig) as evaluated by silver gel analysis (FT = flow-through). Marker: PageRuler™ Prestained Protein Ladder. Data shown for one exemplary experiment. (TIF) [file pone.0321094.s008.tif]

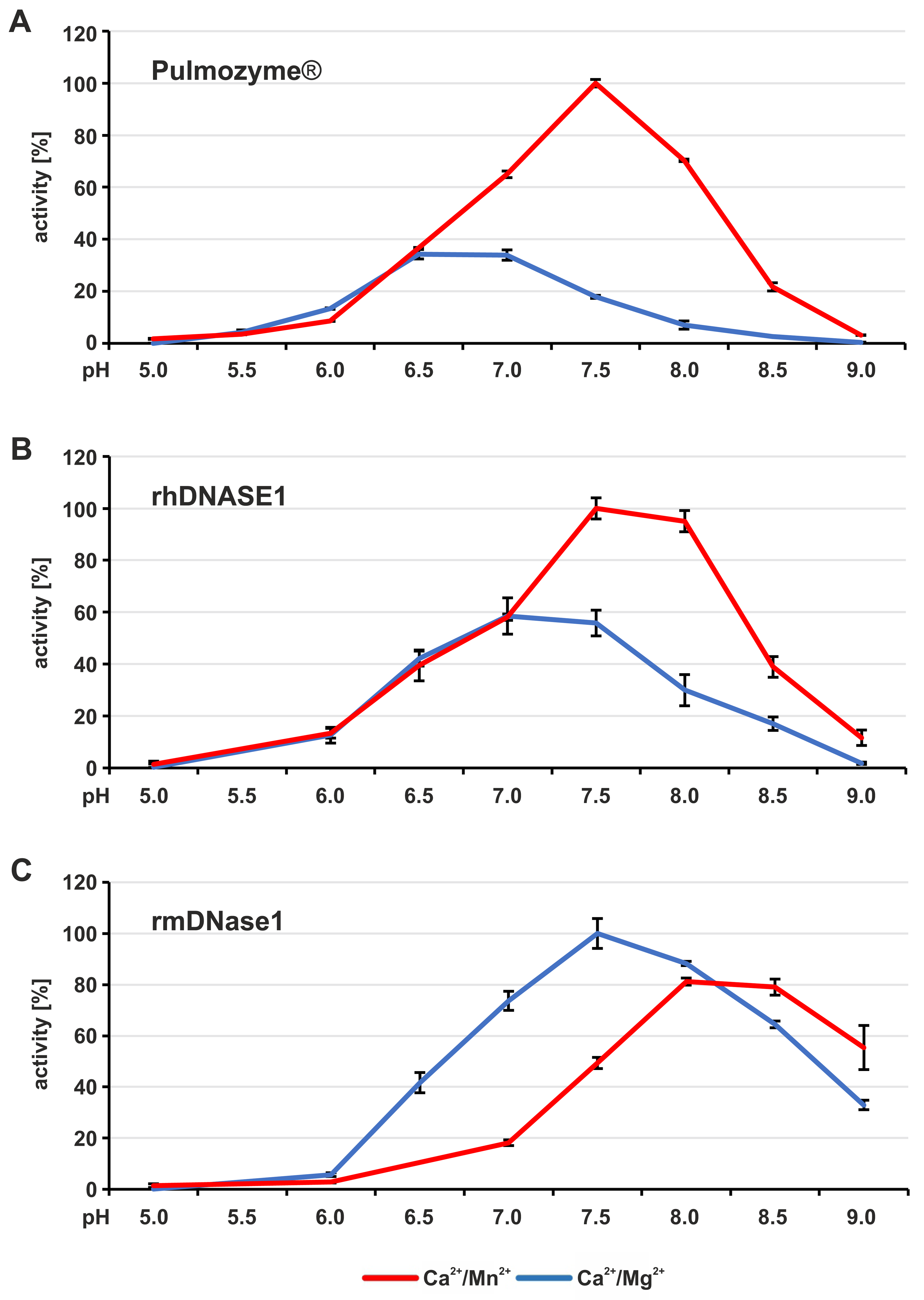

Supplement: S9 Fig — Analysis of the relative DNase1 activity of (A) Pulomzyme®, (B) rhDNASE1, and (C) rmDNase1 with the HCA at different pH in the presence of 0.1 mM CaCl2 combined with either 1 mM MgCl2 or MnCl2. The highest activity measured for each nuclease was set 100%. Pulmozyme® and rhDNASE1 produced in P. pastoris behave similarly. Compared to Pulmozyme® and rhDNASE1, the pH optimum of rmDNase1 is shifted by half a value to the basic direction independently of the co-ion used in addition to Ca2 + . Data shown for one exemplary experiment: mean of each two DNase1 batches measured at least in duplicate. (TIF) [file pone.0321094.s009.tif]

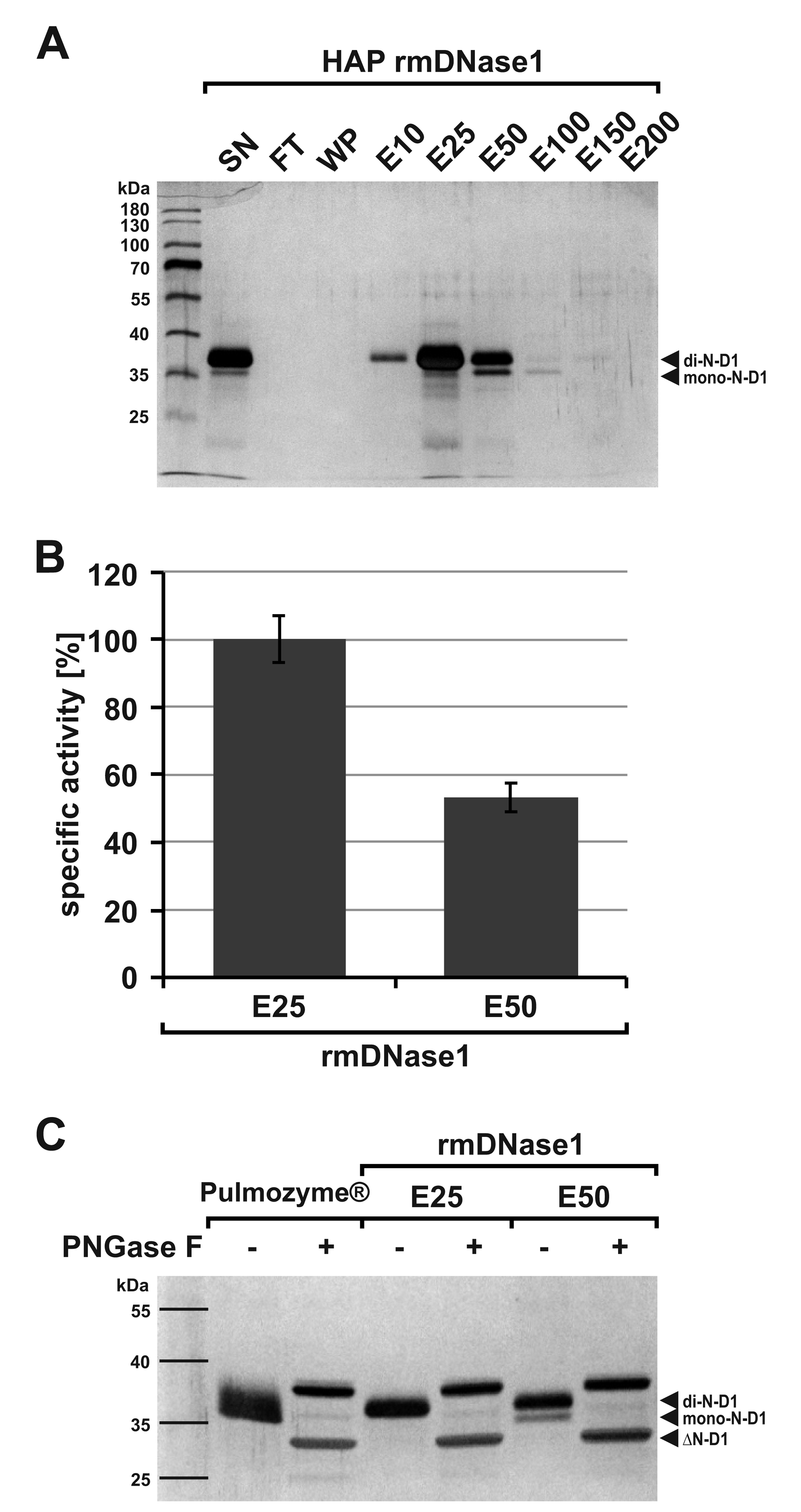

Supplement: S10 Fig — (A) Elution profile of HAP chromatography with rmDNase1 after P-100 gel-filtration as evaluated by silver gel analysis. In contrast to the experiment presented in S7 Fig, an elution step with 25 mM sodium phosphate, pH 6.8 (E25) was included. The eluate E25 contained only di-N-glycosylated rmDNase1 whereas E50 was a mixture of mono- and di-N-glycosylated rmDNase1. (B) Specific activity of rmDNase1 in E25 and E50 as determined by HCA pointing to a reduced activity of the mono-N-glycosylated isoform as evaluated by the difference between the pure di-N-glycosylated E25 vs. the mixed E50 eluate (p = 0.0147). (C) Evaluation of the N-glycosylation pattern and amount of rmDNase1 present in E25 and E50 compared to Pulmozyme®. Each 1 µg protein was treated with PNGase F compared to its untreated control and analyzed by a silver gel. Marker: PageRuler™ Prestained Protein Ladder. Data shown for one exemplary experiment. (TIF) [file pone.0321094.s010.tif]

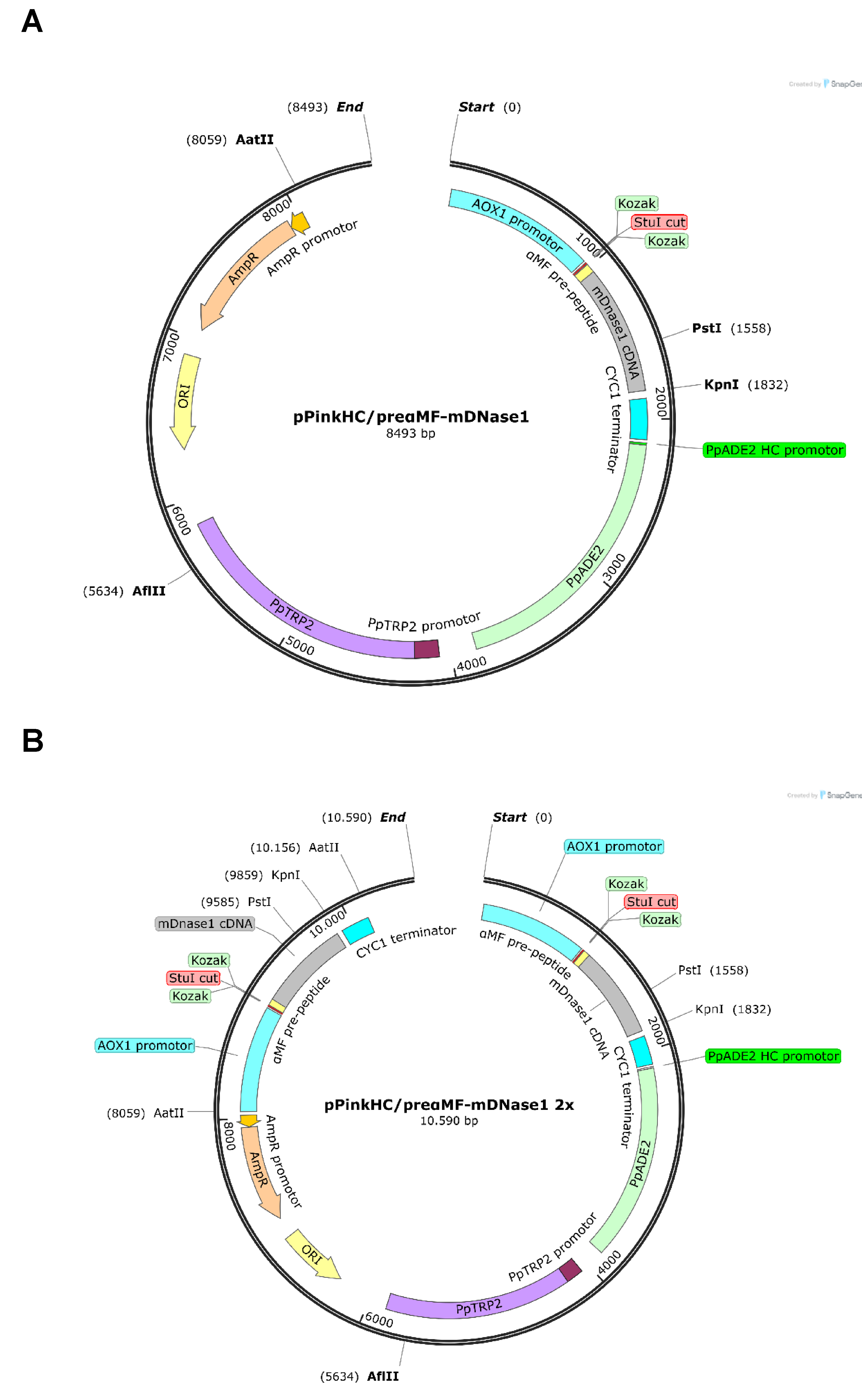

Supplement: S2 File — Vector maps of pPinkHC/preαMF-mDNase1. Maps of vector pPinkHC/preαMF-mDNase1 with a (A) single and (B) double (2x) expression cassette used in this study. Comparable vectors were employed for hDNASE1. Essential features are labeled. The transcription direction of both cassettes is identical. Vectors were linearized by AflII cleavage prior to transformation. (TIF) [file pone.0321094.s012.tif]
