# Supplementary material for: Different results despite high homology: Comparative expression of human and murine DNase1 in Pichia pastoris
Source: PLoS One. 2025 Apr 29;20(4):e0321094. doi: 10.1371/journal.pone.0321094 (PMC12040185; doi:10.1371/journal.pone.0321094)
Supplement: S3 File — The nucleotide sequence for mature hDNASE1 without its natural N-terminal signal sequence of 22 amino acids was taken from GenBank (Acc. No. NM_005223) and codons occurring with a frequency of lower than 25% in P. pastoris were manually revised to the most used ones to generate a codon optimized (co) hDNASE1co cDNA [91]. (PDF) [file pone.0321094.s013.pdf]

S3 File

Wildtype human DNASE1 (hDNASE1), NCBI Reference Sequence: NM_005223.4

CTGAAGATCGCAGCCTTCAACATCCAGACATTTGGGGAGACCAAGATGTCCAATGCCACC 60

CTCGTCAGCTACATTGTGCAGATCCTGAGCCGCTATGACATCGCCCTGGTCCAGGAGGTC 120

AGAGACAGCCACCTGACTGCCGTGGGGAAGCTGCTGGACAACCTCAATCAGGATGCACCA 180

GACACCTATCACTACGTGGTCAGTGAGCCACTGGGACGGAACAGCTATAAGGAGCGCTAC 240

CTGTTCGTGTACAGGCCTGACCAGGTGTCTGCGGTGGACAGCTACTACTACGATGATGGC 300

TGCGAGCCCTGCGGGAACGACACCTTCAACCGAGAGCCAGCCATTGTCAGGTTCTTCTCC 360

CGGTTCACAGAGGTCAGGGAGTTTGCCATTGTTCCCCTGCATGCGGCCCCGGGGGACGCA 420

GTAGCCGAGATCGACGCTCTCTATGACGTCTACCTGGATGTCCAAGAGAAATGGGGCTTG 480

GAGGACGTCATGTTGATGGGCGACTTCAATGCGGGCTGCAGCTATGTGAGACCCTCCCAG 540

TGGTCATCCATCCGCCTGTGGACAAGCCCCACCTTCCAGTGGCTGATCCCCGACAGCGCT 600

GACACCACAGCTACACCCACGCACTGTGCCTATGACAGGATCGTGGTTGCAGGGATGCTG 660

CTCCGAGGCGCCGTTGTTCCCGACTCGGCTCTTCCCTTTAACTTCCAGGCTGCCTATGGC 720

CTGAGTGACCAACTGGCCCAAGCCATCAGTGACCACTATCCAGTGGAGGTGATGCTGAAG 780

TGA 783

Codon optimized human DNASE1 (hDNASE1^{co})

CTGAAGATCGCAGCCTTCAACATCCAGACATTTGGTGAGACCAAGATGTCCAATGCCACC 60

TTGGTCAGCTACATTGTGCAGATCCTGAGCAGATATGACATCGCCCTGGTCCAGGAGGTC 120

AGAGACAGCCACCTGACTGCCGTGGGTAAGCTGCTGGACAACTTGAATCAGGATGCACCA 180

GACACCTATCACTACGTGGTCAGTGAGCCACTGGGAAGAAACAGCTATAAGGAGAGATAC 240

CTGTTCGTGTACAGGCCTGACCAGGTGTCTGCTGTGGACAGCTACTACTACGATGATGGC 300

TGCGAGCCCTGCGGTAACGACACCTTCAACAGAGAGCCAGCCATTGTCAGGTTCTTCTCC 360

AGATTCACAGAGGTCAGGGAGTTTGCCATTGTTCCCCTGCATGCTGCCCCAGGTGACGCA 420

GTAGCCGAGATCGACGCTTTGTATGACGTTTACCTGGATGTCCAAGAGAAATGGGGCTTG 480

GAGGACGTTATGTTGATGGGCGACTTCAATGCTGGCTGCAGCTATGTGAGACCCTCCCAG 540

TGGTCATCCATCAGACTGTGGACAAGCCCCACCTTCCAGTGGCTGATCCCCGACAGCGCT 600

GACACCACAGCTACACCCACGCACTGTGCCTATGACAGGATCGTGGTTGCAGGTATGCTG 660

TTGAGAGGCGCCGTTGTTCCCGACTCGGCTCTTCCCTTTAACTTCCAGGCTGCCTATGGC 720

CTGAGTGACCAACTGGCCCAAGCCATCAGTGACCACTATCCAGTGGAGGTGATGCTGAAG 780

TGA 783
